# Back Accumulation of Diffusive Gradients in Thin-Films Devices with a Stack of Resin Discs To Assess Availability of Metal Cations to Biota in Natural Waters

**DOI:** 10.1021/acs.est.3c00799

**Published:** 2023-05-15

**Authors:** Jordi Sans-Duñó, Joan Cecilia, Josep Galceran, Jaume Puy, Willy Baeyens, Yue Gao

**Affiliations:** † Departament de Química, Universitat de Lleida, and AGROTECNIO-CERCA, Rovira Roure 191, Lleida, Catalonia 25198, Spain; ‡ Analytical, Environmental and Geochemical (AMGC), Vrije Universiteit Brussel (VUB), Pleinlaan 2, Brussels B-1050, Belgium; § Departament de Matemàtica, Universitat de Lleida, and AGROTECNIO-CERCA, Rovira Roure 191, Lleida, Catalonia 25198, Spain

**Keywords:** DGT, back accumulation, stack of resins, dissociation rate constant (*k*_*d*_), speciation, natural waters

## Abstract

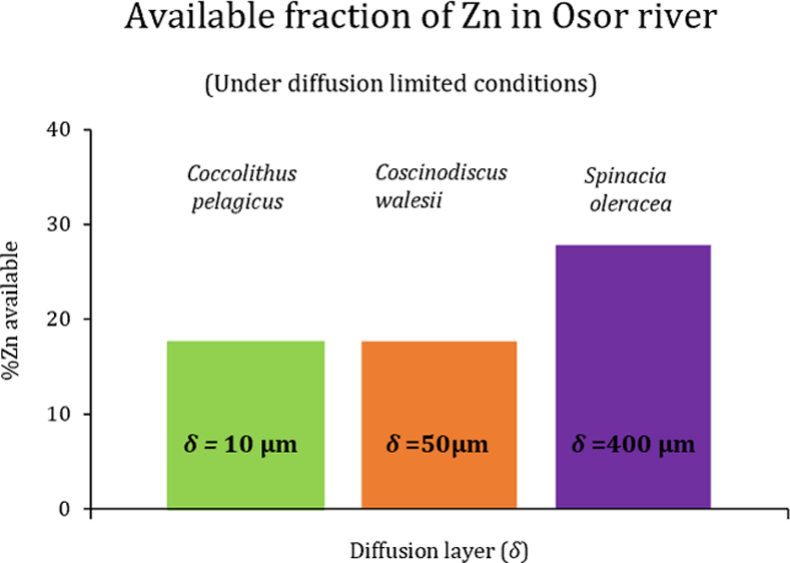

Determining species, concentrations, and physicochemical parameters in natural waters is key to improve our understanding of the functioning of these ecosystems. Diffusive Gradients in Thin-films (DGT) devices with different thicknesses of the resin or of the diffusive disc can be used to collect independent information on relevant parameters. In particular, DGT devices with a stack of two resin discs offer a simple way to determine dissociation rate constants of metal complexes from the accumulation of the target metal in the back resin disc. In this work, simple approximate expressions for the determination of the dissociation rate constant are reported and applied to a model Ni nitrilotriacetic complex as well as to Zn complexes in the Mediterranean Osor stream. Once the physicochemical parameters are known, one can plot the labile fraction of the metal complexes in terms of the thickness of the diffusion domain. These plots reveal a strong dependence on the nature of complexes as well as on the characteristics of the diffusion domain, and they are of high interest as predictors of availability to biota whose uptake is limited by diffusion.

## Abstract 2

A method to determine dissociate rate constants of metal complexes and to estimate metal bioavailability in environmental waters is reported.

## 1 Introduction

Micronutrients such as Fe, Co, Zn, or Mn are governing and limiting autotrophic productivity in high nutrient low chlorophyll (HNLC) areas in the ocean. However, the very complex cellular machinery of phytoplankton species for resource acquisition and for growth makes it necessary to dwell on the concept of “nutrient availability” because trace metals can exist in different forms in the ocean (free ion, weak and strong inorganic and organic complexes, and colloids), and they are not all bioavailable. The same reasoning as for the micronutrients can be held for toxic trace metals. Knowledge of trace-metal availability is, thus, of crucial importance to understand key processes, such as phytoplankton growth or intoxication of organisms in natural aquatic systems. Availability of metal cations to microorganisms or chemical sensors results from a set of physicochemical and biological phenomena that include at least the mobility and kinetics of dissociation of the species present in the aquatic medium and the internalization of the metal ion, whenever the free metal ion is the only one chemical species able to be internalized, i.e., transported through the cell membrane.^1−4^

The consumption of the metal cation by internalization leads to the development of a free metal concentration profile. All equilibrium processes involving the metal cation are, then, shifted to buffer the metal consumption, and both the kinetics of dissociation and the transport determine the contribution of each species to the accumulation. For trace metals in natural waters, total metal concentrations are well below those of simple or macromolecular ligands and, then, the accumulation is mainly dependent on the properties of the complexes.^5^ When transport is the rate-limiting step in the uptake process, the system is called labile, while, when dissociation is the rate-limiting step, the accumulation is under kinetic control.^6^ Only when the internalization is the rate-limiting step, the concentration profile of all metal species is almost flat and availability is given by the free metal concentration as predicted by the biotic ligand model (BLM).^7^

**Figure 1:**
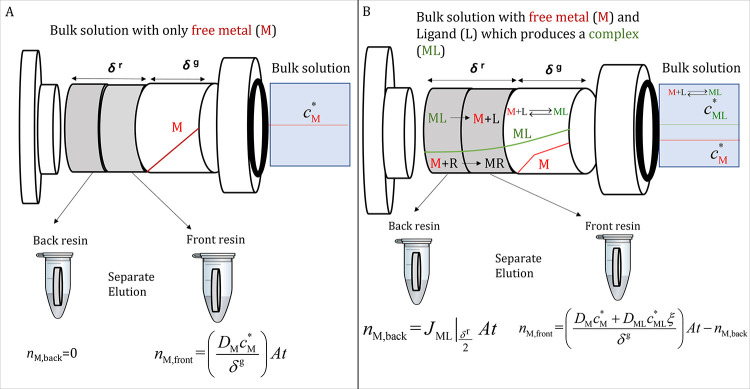
Schematic representation of a DGT device with a diffusive gel (of thickness δ^g^) and two resin discs (each one of thickness δ^r^/2) deployed in a solution with only free metal (M) at a concentration *c*_M_^*^ (Panel A) or in a solution that also contains M, a ligand (L), and a partially labile complex (ML) (Panel B) with concentrations *c*_M_^*^, *c*_L_^*^, and *c*_ML_^*^, respectively. The figure also depicts schematic concentration profiles of M (red line) and ML (green line) in excess of ligand conditions and steady-state.

In laminar conditions, sufficiently close to the consuming surface of the living organism or of the chemical sensor (*x* = δ^r^, in Figure 1), the arrival of the metal species takes place by diffusion. Beyond the diffusion domain (i.e., *x* > δ^r^ + δ^g^), bulk concentrations of free metal and complexes are usually assumed as fulfilling equilibrium conditions, unless experimental measurements lead to other considerations.

The thickness of the diffusion domain defines a timescale window that needs to fit the dissociation of the complex to contribute to its availability.^8^ Thus, availability is sensor dependent so that the metal flux, measured by different analytical techniques such as Diffusive Gradients in Thin-films (DGT) and anodic stripping voltammetry or by several measurements of only one technique with configurations where the thickness of the diffusion domain varies, can be used to obtain a labile concentration signature that characterizes the natural water.^9,10^

The contribution of complexes to the metal flux either individually or collectively (the global set of complexes) can be quantified with the so-called lability degree.^11−13^

The lability degree indicates the ratio of the current contribution of a complex (or of all the complexes present in the system when the global lability degree is considered) to the metal flux over the maximum contribution of this/these complex(es) that could be reached if dissociation was so fast that complexes were in equilibrium with the free metal at all times and relevant spatial positions.^6,14−16^

DGT devices with a stack of resin discs allow one to access the metal accumulation in each resin disc of the stack by separate elution of each one (see Figure 1).^17,18^ In this work, DGT devices with a stack of two resin discs will be used. The top or front resin disc is in contact with the gel disc, and it accumulates the free metal arriving by diffusion from the gel disc plus the metal released by dissociation of complexes in this front resin disc.^19^ Further away from the sampled solution, we find the bottom or back resin disc where the whole metal accumulated comes from the dissociation of partially labile complexes provided that the front resin disc is far from saturation, the binding agent is well dispersed in the front resin disc (i.e., the free metal cannot diffuse and cross any resin disc without getting bound), and the formation of ternary complexes with the resin sites is negligible. Indeed, since both, the diffusive and resin gels, share common pore sizes, complexes able to diffuse through the diffusive gel disc can also penetrate the front resin disc and the back one where they will dissociate. A non-negligible presence of mass of a target metal in the back resin disc indicates, then, the presence of partially labile complexes. The use of DGT devices with a stack of resin discs is, then, relevant to reveal the presence of partially labile complexes, as well as to obtain the dissociation characteristics of these complexes in an easy way.

In this work, a more general expression for the back accumulation is reported without neglecting the accumulation in the front resin disc due to the flux of free metal as done previously.^20^ Simple criteria are discussed to decide in which cases the simplified formula yields accurate values of the dissociation rate constant. This discussion suggests that the settling of the resin beads could also be relevant in the back accumulation. Accordingly, an analytical expression for the percentage of back accumulation that takes into account the heterogeneity of the resin bead distribution (due to the settling process during casting) in the DGT resin discs is reported. The procedure is applied to determine the dissociation rate constant of the well-defined Ni nitrilotriacetic acid (NTA) complex, NiNTA. The back accumulation in DGT devices deployed in a Mediterranean river is also used to determine an effective dissociation rate constant for a pool of inorganic zinc complexes in this freshwater. In both cases, dissociation rate constants obtained from the back resin accumulation are compared with values that can be obtained from the total accumulation, since accumulation is also impacted by the lability of complexes.

In this framework, once the dissociation rate constant is known, the “effective” metal concentration seen by biota in the media can be predicted as a function of the thickness of the diffusion domain developed around the surface of the biota driven by the uptake process.

## 2 Theoretical Framework: Dissociation Rate Constant and Accumulation in DGT Devices

### 2.1 Assuming Perfect Dispersion of the Resin Beads in the Resin Disc

DGT is a dynamic technique that measures, for a certain deployment time, the metal accumulation in a resin disc. On top of the resin disc, a diffusive gel is used to define a diffusive domain where the metal species, coming from the aqueous sample, travel by diffusion toward the resin disc. When the binding to the resin is fast and strong, after a short time, the accumulation takes place in a quasi-steady-state regime. The flux in the DGT device can be computed as the accumulation per unit of time and surface, and it depends on speciation, mobilities, labilities, and other factors such as pH and ionic strength. For a metal M which only forms a complex, ML, in a given medium, the total accumulation can be written as:^21^
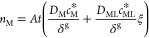
1where *n*_M_ is the accumulation (moles) of M at a certain deployment time *t*, *c*_M_^*^ and *c*_ML_^*^ are the bulk equilibrium concentrations of the free metal and complex in the sample solution, *D*_M_ and *D*_ML_ are the respective diffusion coefficients, δ^g^ is the thickness of the diffusive gel disc plus that of the diffusive boundary layer (DBL, the extension of the diffusion domain into the solution, omitted in Figure 1 for the sake of clarity), *A* labels the area of the DGT device, and ξ is the lability degree of the complex. The lability degree in terms of the physicochemical parameters, for one complex in excess ligand conditions, can be written as:^21,22^
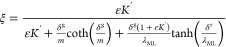
2where  describes the ratio of diffusivities,  is the excess-ligand conditional stability constant, δ^r^ is the thickness of the resin layer, λ_ML_ is related to the thickness of complex penetration into the resin layer before full dissociation,
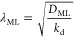
3and *m* is related to the reaction layer thickness in the diffusive gel disc:
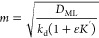
4and *k*_d_ is the dissociation rate constant.

The lability of complexes in DGT devices tends to be higher than in other sensors due to the (relatively large) thickness of the diffusive gel, of the order of 10^–3^ m, and to the penetration of complexes in the resin disc.^8^ Indeed, the reaction layer, where there is net dissociation of the complexes, starts at the neighborhood of the resin-gel interface and penetrates into the resin domain where free metal concentration is negligible due to the typical fast and strong binding of the metal to the resin (perfect sink conditions).^21^

In a laboratory experiment, when the speciation and diffusion coefficients are known, eq 1 can be used to determine the lability degree and, in a further step, by solving eq 2 to determine the dissociation rate constant of the complex.

The calculation of the dissociation rate constant from the accumulation can be extended to systems with a mixture of complexes like those in natural media. In presence of a set of complexes, the second term of the r.h.s. of eq 1 would contain a summation for all the complexes. The knowledge of relationships between lability degrees, sensor characteristics, and physicochemical parameters of the chemical species^21,22^ opens the way to use the measurements of a set of sensors (equal or larger than the number of unknowns) to determine physicochemical parameters of the system as well as to predict the availability to microorganisms. In this case, a set of unknown kinetic dissociation constants and, accordingly, a set of DGT devices with different configurations in terms of thickness of the diffusive or the resin discs are required to write a set of equations determining the set of dissociation rate constants.^17,18,23−25^ Mobilities can also be included in the set of unknowns. Nonetheless, the detailed application of this procedure to natural waters is still unapproachable, unless a reduced number of pools of complexes sharing similar mobilities and labilities could be defined to simplify the system. In any case, this method allows a step further in the understanding of the behavior of metal complexes in natural systems.

In this work, another independent strategy to obtain the dissociation rate constants of complexes is proposed.

When DGT devices with a stack of two resin discs are used (see Figure 1 for a scheme), the metal concentration drops to zero at the resin-diffusive gel interface (perfect sink) and remains negligible along all the resin discs whenever we are far from saturation. Thus, when there is only metal in the solution (Panel A of Figure 1), the metal does not reach the back resin disc. However, in presence of complexes (Panel B of Figure 1), the decreasing concentration of the complex toward the bottom of the DGT device indicates a flux of complex in this direction that, in steady state, needs to dissociate to avoid complex accumulation. Since there is no free metal in both resin discs (the M profile is flat (no diffusion) and equal to zero), all the metal released by dissociation of the partially labile complex ML is retained in the corresponding resin disc where dissociation takes place. Thus, in the back resin disc, only metal from the local dissociation of partially labile complexes is expected to be found whenever ternary complexes are negligible. Once these discs are eluted separately, the mass in the back resin disc informs on the presence of partially labile complexes as well as on their dissociation rate constants. Notice that the resin and diffusive gel layers drawn in Figure 1 are magnified to help in the visualization of the processes and concentration profiles.

Let us define the percentage of back accumulation, *B*, as the moles of metal accumulated in the back resin divided by the total moles accumulated in all resin layers of the DGT.

In steady state, the accumulation in a back resin per unit of time can be computed as the total metal flux crossing the plane *x* = *δ*^r^/2 (which is just due to the complex ML), while the total accumulation per unit of time (in both resins) equals to the total metal flux crossing *x* = *δ*^r^. Accordingly
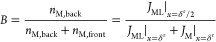
5

Assuming perfect sink conditions, excess of ligand and steady-state concentrations, *B* can be written as:^20^
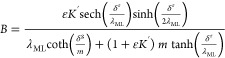
6as derived also in Section S4, eq S89, of the Supplementary Information (SI).

Eq 6 relates *B* with the dissociation rate constant of a complex allowing the determination of *k*_d_ by numerical solution of this equation (see SI, Section S5) once *B* is experimentally known.

Additionally, when ε*K*′ ≫ 1, the transport of the metal toward the resin mainly takes place through the complex. In these conditions, it has been shown that most of the metal accumulated comes from the dissociation of complexes inside the resin domain,^19^ i.e., . With this approximation, eq 6 becomes:
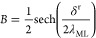
7and, accordingly
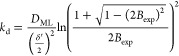
8

Eq 8 leads to the determination of *k*_d_ from an experimental value of *B* (*B*_exp_) provided that *D*_ML_ is known.

**Figure 2:**
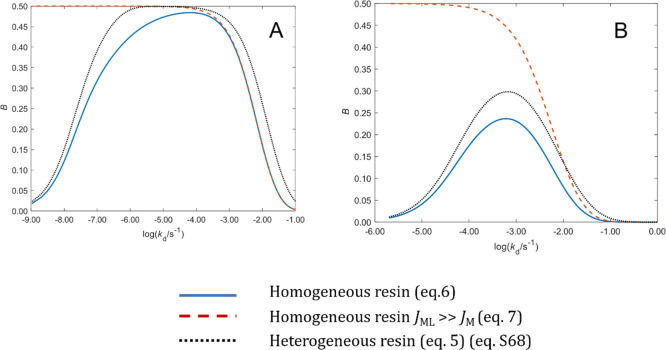
Percentage of back accumulation against the dissociation rate constant (*k*_d_) while keeping the ratio *k*_a_/*k*_d_ constant. (Panel A) *K′* = 3.63 × 10^4^, *D*_Ni_ = 7.07 × 10^–10^ m s^–2^, *ε* = 0.7, *δ*^g^ = 8.54 × 10^–4^ m, *δ*^r^ = 8 × 10^–4^ m, and *k*_d,E_ = 7.7 × 10^–6^ s^–1^, and (Panel B) *K′* = 4.70, *D*_Zn_ = 4.20 × 10^–10^ m s^–2^, ε = 1.0, *δ*^g^ = 1.079 × 10^–3^ m, and *δ*^r^ = 8 × 10^–4^ m. The blue line stands for eq 6 that considers a homogeneous distribution of beads in the resin disc. The dashed red line is given by eq 7, and the dotted black line is given by eq S78 in the SI assuming a heterogeneous distribution of the resin beads.

Figure 2A plots *B* computed with eqs 6 and 7 vs the dissociation rate constant (*k*_d_) for a case with *εK*′ = 2.54 × 10^4^. According to the figure, for *k*_d_ > 10^–3^ s^–1^, there is a good agreement between eqs 6 and 7. However, as *k*_d_ decreases, both equations diverge, and while eq 7 tends to *B* = 0.5 regardless of the *k*_d_-value, *B* decreases according to eq 6 without reaching the value 0.5. This is a fundamental difference between both expressions that comes from neglecting the flux entering to the resin as free metal in the derivation of eq 7. Indeed, for any *εK*′ value, a low enough lability of the complex will render negligible the dissociation inside the resin compared to the flux entering to the resin as free metal, so the use of eq 6 would be required.

As ε*K*′ decreases, see Figure 2B where *εK*′ = 4.70 in contrast to *εK*′ = 2.54 × 10^4^ of Panel A, the agreement between eqs 6 and 7 is restricted to higher *k*_d_-values (*k*_d_ > 10^–1^ s^–1^ in Figure 2B) because of the increase of the metal flux .

### 2.2 Influence of the Settling of the Resin Beads in *B*

As it is well documented, the resin beads settle during casting, so that both large surfaces of the resin disc are not equal.^26^ Schemes illustrating this heterogeneity are included for instance in refs (27) and (28).

A small roughness indicates the side of the resin disc that should be placed in contact with the diffusive gel when the DGT device is mounted. The settling of the resin beads can be clearly appreciated in Figure S1, a picture obtained with a microscope of the resin gel. The influence of this heterogeneity on the accumulation has been studied in ref (20).

The settling influences accumulations when a DGT device with a stack of resin discs is used, especially in the presence of partially labile complexes. In the supplementary information, we provide details on the calculation of *B* considering this effect with eq S78, to which we are going to refer as the heterogeneous model.

Figure 2 (both panels) also plots *B* computed with eq S78 considering that only half of the volume of each resin disc contains resin beads as suggested by the picture of the resin reported in Figure S1. This is a reasonable assumption given the size of the beads (100 μm) and the thickness of the disc. Notice that for a fixed *k*_d_, the value of *B* computed with eq S78 is greater than the value resulting from eq 6 which corresponds to a homogeneous distribution of the resin beads. This difference can be understood from the observation of the concentration profiles of free metal and complex in a heterogeneous resin depicted in Figure S4. The percentage of metal accumulation in the front resin disc is higher in the homogeneous resin since the front resin disc collects all the metal released in its volume, while in the heterogeneous resin, part of the metal released in the front resin disc volume is collected in the back resin disc.

As discussed in ref (20), maximum discrepancies in the accumulation between the homogeneous and the heterogeneous models appear for lability degrees close to 0.25. Regarding back accumulation, Figure 2 indicates that differences between eqs 6 and S78 are negligible for small *B* values since the system is either very inert or very labile and there is practically no dissociation of the complex in the resin domain. For intermediate *B* values, the difference, according to Figure 2, is quite independent of *K*′ and close to 0.3 log units especially at the right side of the maximum that corresponds to systems where complexes are more labile and relevant for the accumulation. Thus, *k*_d_ calculated with the heterogeneous model is close to a factor of 2 higher than the *k*_d_ calculated with the homogeneous model when we are on the right side of the maximum (or half of the *k*_d_ calculated with the homogeneous model when we are on the left of the maximum) as expected from the argument that a reduction of one-half in the volume of dissociation requires a double kinetic dissociation constant to release a given amount of free metal. For *B* values close to the maximum, the differences in *k*_d_ recovered from both models increase and the homogeneous model may even be unable to justify *B*_exp_. A plot of the maximum *B* value calculated with the homogeneous model vs *K*′ can be seen in Figure S5. *B*_exp_ values higher or close to the maximum *B* of the homogeneous model require the use of the heterogeneous model to recover *k*_d_-values.

In the following sections, we are going to apply eqs 5–7 for the determination of the kinetic dissociation rate constants of NiNTA complexes as well as to Zn complexes in the freshwater Osor, a small tributary of the Terriver in the northeast of Spain.

## 3 Methodology

### 3.1 Experimental Section

#### 3.1.1 Laboratory Experiments: Ni Accumulation in DGT Devices in the Presence of NTA at pH = 7.5

Experimental details are described elsewhere.^22^ Briefly, DGT holders (piston type, 2 cm diameter window) and polyacrylamide gel discs (diffusive disc, 0.8 mm thick, Chelex resin disc, 0.4 mm thick) from DGT Research Ltd. were used. The 5 L polyethylene exposure chamber was kept at 25 °C using a thermostat and stirred at 240 rpm. DGT devices were deployed per triplicate in solutions of 0.5 mol L^−1^ NaNO_3_ (Merck, Suprapur) and 10^–5^ mol L^–1^ Cd and Co and 2.5 × 10^–5^ mol L^–1^ Ni (prepared from 10^–2^ mol L^–1^ stock solutions of nitrate salts Cd(NO_3_)_2_·3H_2_O, Co(NO_3_)_2_^.^6H_2_O, Zn(NO_3_)_2_^.^6H_2_O, and Ni(NO_3_)_2_^.^6H_2_O) and 8 × 10^–5^ mol L^–1^ NTA (Fluka, analytical grade) during 24 h at pH = 7.5 (1 mmol L^−1^ MOPS, Sigma-Aldrich). In addition to standard DGT devices composed of one resin layer and one gel layer, DGT devices with two resins layers (labeled as 2R1G devices) were also used. The front and the back resin discs were eluted separately.

#### 3.1.2 DGT Accumulation in the Osor Stream

The Osor stream is a small tributary of the Terriver located in Girona province (Catalonia) in the northeast of Spain whose surface waters are impacted by former mining activities of F–Ba–Pb–Zn ore.^29,30^

Our sampling strategy was mainly designed to assess the speciation of Zn by deploying DGT devices in this freshwater as described elsewhere.^10^ At the sampling site, coordinates 41° 57′14″ N, 2° 35′58″ E, a set of parallelepiped structures was fixed with the purpose of holding the DGT devices parallel to the river flow. During March 10, 2020, and March 11, 2020, *in situ* measurements of a set of physical parameters were taken with an Orion Star A329 pH/ISE/Conductivity/RDO/DO Meter and samples were collected and prepared *in situ* by filtering 9.98 mL of stream water through a 0.45 μm syringe filter and mixing it with 20 μL of HNO_3_ 70%. These samples were analyzed for total Zn using an inductively coupled plasma mass spectrometer (ICP-MS, 7700x, Agilent Technologies, Inc.). The electroanalytical technique absence of gradients and Nernstian equilibrium stripping (AGNES) was applied to the last grab sample from March 11, 2020^10^ to determine the free Zn concentration in the water. Main anions and cations were measured with ion chromatography (IC), alkalinity of this last grab sample was analyzed in the Scientific and Technical Services of the Catalan Institute for Water Research (ICRA), and dissolved organic carbon (DOC) was analyzed at “Serveis Cientificotècnics” of the Universitat de Lleida.

### 3.2 Determination of *K′*

For the well-defined NiNTA complex, the speciation code VMINTEQ allows a detailed prediction of the species and concentrations in solution under conditions reported in Section 3.1 so that *K*_NiNTA_ can be computed by just dividing the equilibrium concentration of the complex (*c*_NiNTA_^*^) by the free Ni (*c*_Ni_^*^) resulting in *K′* = 3.63 × 10^4^.

In the Osor stream, *c*_T,Zn_ measured with ICP-MS was 633 nmol L^–1^ and *c*_Zn_^*^, obtained from AGNES, was 111 nmol L^–1^.^10^ Other total concentrations are listed in Section S10, Table S1, of the Supporting Information.

Speciation of Zn in the Osor stream can also be estimated using VMINTEQ. The prediction is that zinc is mainly present in the freshwater as free zinc (*c*_Zn_^*^) 192 nmol L^–1^ (in agreement with the concentration obtained by AGNES result) and a set of inorganic complexes (ZnCO_3(aq)_, Zn(OH)_2_, ZnOH^+^, etc.) with concentrations of 78.6, 287, and 22.5 nmol L^–1^, respectively. Complexes of Zn with dissolved organic matter, computed with default parameters of NICA-DONNAN,^31^ have a concentration of 8.21 nmol L^–1^ which represents around 1% of *c*_T,Zn_ so that from now on, we are going to neglect the Zn bound to dissolved organic matter.

Assuming that the inorganic complexes could be treated as a pool with a common diffusion coefficient, *D*_ZnL_ = *D*_Zn_, the concentration of this pool can be estimated as *c*_ZnL_^*^ = *c*_T, Zn_ – *c*_Zn_^*^ and *K′* = 4.70 of this pool was straightforwardly obtained by dividing *c*_ZnL_^*^ by *c*_Zn_^*^ (111 nmol L^–1^).

### 3.3 Determination of *k*_d_ from *B*

The use of the limiting expression (eq 8), when applicable, allows a straightforward determination of *k*_d_, while when eq 6 needs to be used, an iterative procedure can be applied.

In order to have a simple criterion for the plausibility of the application of eq 8, we need to explore the fulfillment of the approximation:
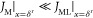
9on which the limiting expression (eq 8) relies. The reaction layer concept^32^ applied to the DGT devices can provide approximate expressions for  and .^19^ As detailed in the SI (see eq S93), with these expressions, the inequality  reduces to
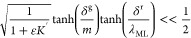
10whose fulfillment validates the *k*_d_-value obtained with the limiting expression (eq 8). Conversely, if eq 10 is not satisfied, a better approximation for *k*_d_ should be sought using eq 6 instead of eq 8.

In order to check whether the heterogeneity of the resin beads distribution is relevant for the determination of *k*_d_, we can examine the inequality *B*_exp_ < *B*_max_ (where *B*_max_ is the maximum back percentage that can be reached in a stack of two homogeneous resin discs). A plot of *B*_max_ vs *K*′ for the homogeneous resin and typical values of the rest of parameters is given in Figure S5 of the SI. When *B*_exp_ is close to or greater than *B*_max_, then eq S78 is needed to recover accurate *k*_d_ values as discussed in Section 2.2.

When eqs 6 or S78 have to be used, we notice that according to Figure 2, two *k*_d_-values are obtained, corresponding to a more inert or a more labile system. Both *k*_d_-values can be used to compute accumulations (with eqs 1–4) to be compared with the experimental one in order to select the one leading to the closest agreement. See details in Section S5 of the SI.

An excel file ready to generate the solutions of eqs 8, 6, and S78 is provided as SI. See Section S9 in the SI for further instructions. According to the results of the inequalities eq 10 and *B*_exp_ < *B*_max_, the selected value of *k*_d_ appears in a cell with green background.

## 4 Results and Discussion

### 4.1 Determination of *k*_d_ for the NiNTA Complex Using 2R1G DGT Devices

A previous work^22^ showed that while the dissociation in the diffusive gel follows Eigen principles based on a dissociative mechanism, the dissociation reaction of the NiNTA complex (the rest of Ni complexes in solution are fully labile) in the resin layer follows a faster ligand-assisted mechanism. Thus, a dissociation rate constant, *k*_d,E_ (7.7 × 10^–6^ s^–1^),^22^ was assumed in the diffusive gel, where subscript E indicates Eigen mechanism, while a dissociation rate constant, *k*_d,R_, was considered for the dissociation within the resin disc which corresponds to a ligand-assisted exchange mechanism. Actually, both mechanisms compete inside the resin disc, but the higher rate of the ligand-assisted one renders negligible the presence of the Eigen mechanism in the resin domain.

Accordingly, λ_ML_ involves *k*_d_,_R_, while *m* uses *k*_d_,_E_ in eq 2 when the determination of *k*_d_ is done from the lability degree or when it is determined from *B* using eq 6.

As seen in Table 1, using eq 8 and *k*_d,E_ = 7.7 × 10^–6^ s^–1^, a dissociation rate constant *k*_d,R_ of 4.0 × 10^–3^ s^–1^ has been obtained for the partially labile complex NiNTA. When eq 10 is checked, we obtain 3.88 × 10^–3^ ≪ 1/2, indicating that the condition is satisfied. Therefore, the *k*_d_ value computed with eq 8 can be accepted as can be confirmed by comparing this value with that obtained from the complete eq 6, also reported in Table 1, 4.1 × 10^–3^ s^–1^. These values are in good agreement between them and with the result obtained from the lability degree 2.8 × 10^–3^ s^–1^.^33^

**Table 1:** Dissociation Rate Constant Values Determined from the Back Accumulation (with Either the Homogeneous or the Heterogeneous Resin Model)^a^

model	laboratory experiments *k*_d_ NiNTA (s^–1^)	Osor stream *k*_d_ ZnL_Inorg_ (s^–1^)
homogeneous resin and eq 8	4.0(±0.9) × 10^–3^	3(±1) × 10^–3^
homogeneous resin and eq 6	4.1(±0.9) × 10^–3^	undetermined
heterogeneous resin and eq S78 in the SI	8 (±2) × 10^–3^	1.2(±0.6) × 10^–3^
lability degree (ξ), found from eq 1	0.67(±0.01)	0.76(±0.03)
*k*_d_ from lability degree (ξ) and eq 2	2.8(±0.3) × 10^–3^	2.8(±0.7) × 10^–3^

a The parameters needed to calculate *k*_d,R_ (for NiNTA) or *k*_d_ (for Zn in Osor) are specified in Section 3.2 and Figure 2.

As the *B*_exp_ value (0.29 ± 0.03 for NiNTA) is well below the corresponding *B*_max_ value obtained from Figure S5 of the SI for ε*K*′ = 2.54 × 10^4^, we expected that the simplified expression (eq 8) would yield an accurate value (i.e., within the order of magnitude) of the rate dissociation constant of this complex. Indeed, applying the expression for *B* reported in eq S78, *k*_d,R_ results in 8 × 10^–3^ s^–1^, a value just twofold higher than the homogeneous estimation of *k*_d,R_ as expected from Figure 2.

### 4.2 Determination of *k*_d_ for Inorganic Zn Complexes in the Osor Stream

The same procedure (except that a single *k*_d_ is assumed everywhere), applied to the availability of Zn in the stream, leads to a different outcome. The simplified expression eq 8 produces *k*_d_ = 3 × 10^–3^ s^–1^. This value of *k*_d_ in eq 10 yields 0.25, a value smaller than 1/2, but not at the level of orders of magnitude. Nevertheless, this *k*_d_-value cannot be compared with the one obtained from the more complete expression (eq 6) because this equation does not yield any *k*_d_ value with a *B* = 0.29 ± 0.06 for the set of used parameters, indicating that *B*_exp_ value is higher than *B*_max_ = 0.22 reported in Figure S5 of the SI for *K*′ = 4.70. Therefore, in this case, we need to apply the heterogeneous model for the resin layer to obtain a solution for *k*_d_.

As seen in Figure 2, a *B* equal to 0.29 ± 0.06 yields two solutions of eq S78: *k*_d_ = 1.2 × 10^–3^ s^–1^ and *k*_d_ = 3.4 × 10^–4^ s^–1^. For the Osor stream, the selected solution is *k*_d_ = 1.2 × 10^–3^ s^–1^ according to the agreement of the Zn accumulation predicted by eqs 1–4 with the experimental measurement.

The difference between this value (*k*_d_ = 1.2 × 10^–3^ s^–1^) and the one obtained from the lability degree assuming a homogeneous resin, *k*_d_ = 2.8 × 10^–3^ s^–1^, reflects the differences between models as well as the corresponding experimental uncertainties associated to the back and to the total accumulation, respectively.

In conclusion, DGT devices with two resin discs provide complementary information of the system that can be used, at least, to double-check the determination of reaction-rate constants from the lability degree.

### 4.3 Environmental Implications

When internalization is not the rate-limiting step, the free metal develops a concentration profile supported by the dissociation of complexes that depends on the respective diffusion coefficients and the thickness of the diffusion domain. Dynamic analytical techniques can mimic, in this case, the uptake process of some biota becoming suitable for predicting bioavailability. It should be noticed that different dynamic techniques such as DGT, permeation liquid membrane,^34,35^ or stripping voltammetry (including Scanned Stripping ChronoPotentiometry, SSCP) yield different availability measurements in a common sample reflecting different contributions of complexes in agreement to the different diffusion layer thicknesses of each technique.^8^ Likewise, different microorganisms, algae, or biological consumers face different available concentrations in a common sample since they exhibit different diffusion domains. These different domains induce different dissociation degrees of metal complexes, which are a signature of the lability of the pool of complexes in the sample.

While the diffusion domain extends out of the biota membrane, in DGT, complexes can penetrate in the resin disc, so that the diffusion domain encompasses both the diffusive and the resin disc. For a closer resemblance with the biouptake process of biota, once *k*_d_ is determined as explained in this work, we can compute the expected accumulation (*n*_M_) in a DGT device without penetration of complexes by setting δ^r^ = 0 in eqs 1 and 2 as done in ref (10). Defining as usual , a plot of *c*_DGT_ vs δ^g^ computed with δ^r^ = 0 and a given total concentration of a target metal can be understood as the potential maximum “effective” concentration that a microorganism can uptake in the studied solution according to its thickness of the diffusion domain. Notice how this concentration increases as the thickness of the diffusion domain of the organism increases due to a higher contribution of partially labile complexes to the accumulation reaching the total concentration of the metal when all the complexes are labile and with a common diffusion coefficient with the free metal.^36^

**Figure 3:**
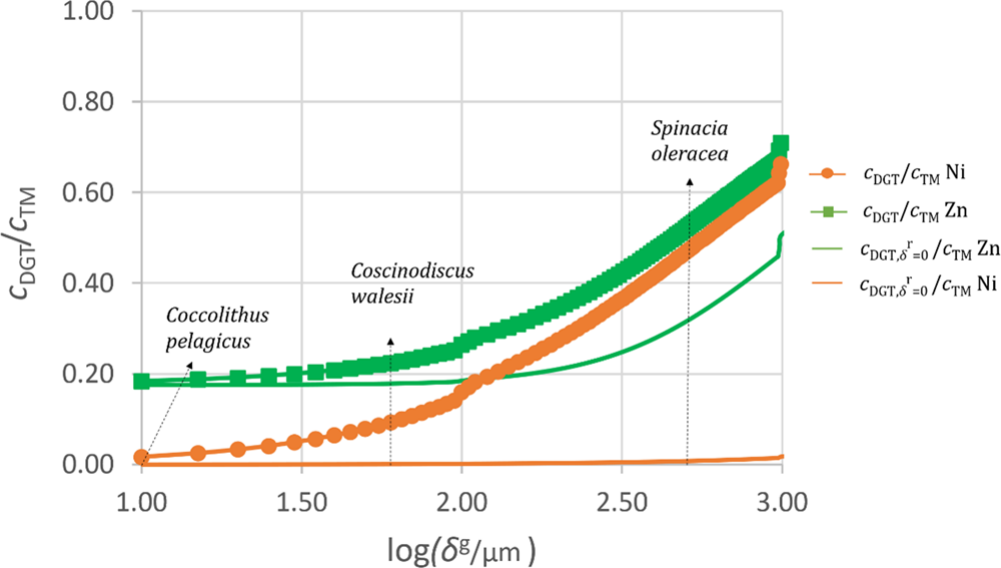
Available DGT concentration as a function of the diffusion thickness for two types of DGT (δ^r^ = 4 × 10^–4^ m and δ^r^ = 0 m) devices and two types of metal complexes (ZnL_Inorg_ of Osor water and NiNTA) for a *c*_T,Zn_ = 6.33 × 10^–4^ mol m^–3^ and *c*_T,Ni_ = 2.5 × 10^–2^ mol m^–3^.

Figure 3 depicts *c*_DGT_ (normalized with respect to the total metal concentration) for Ni in our laboratory solution with NTA as well as *c*_DGT_ for Zn in the Osor stream expected for any biota whose diffusion domain thickness ranges from 10 μm to 1 mm.

In any case, an important decrease of the Ni and Zn availability is seen in the figure from the standard DGT device to a DGT without penetration of complexes in the resin disc.

For the case of Zn, the minimum availability is close to 20% of the total Zn, in agreement with the free Zn concentration (111 nmol L^−1^) in this freshwater, but it reaches a value close to 50% for biota with diffusion domain thickness close to 1 mm (see continuous green line). For example, diatom species such as *Coccolithus pelagicus* or *Coscinodiscus wailesii* are expected to show an availability of 20% of the total concentration because their radius is between 2–10 μm and 50 μm, respectively, which is also the size of their diffusion domain.^37−39^ Ref (40) used a diffusion layer length of 400 μm to determine the flux available to *Spinacia oleracea* grown in hydroponic media. According to Figure 3, it would face an availability of 30% of the total Zn concentration of the Osor water.

For the case of Ni, as complexes with NTA are strong, biota with a diffusion domain thickness from 10 to 1000 μm would face an effective Ni concentration lower than 1% of the total Ni (see continuous orange line). Thus, *S. oleracea* in the same solution would face the same “effective” concentration of Ni as *C. pelagicus* or *C. wailesii* even though its diffusion thickness is 40 times greater than the one of *C. pelagicus*.

The building of figures similar to Figure 3 can be of high environmental interest for any target element in natural waters. In the present case, this process has been facilitated by the determination of the dissociation kinetic constants of the complexes. Obviously, limitations in this task come from the measurement of the low free metal concentrations in some waters, the identification of the ligands, the determination of the physicochemical parameters, and the fact that we lack knowledge about the presence of functional ligands on the outer membrane of the organisms. The use of DGT devices with different thicknesses of the diffusive and resin discs, as well as devices with a stack of resin discs, can be helpful to improve our understanding of the lability and availability of metal complexes in natural waters.

## Supporting Information Available

Graphical description of the heterogeneous and homogeneous resin models; derivation of a solution for *c*_M_ and *c*_ML_ for heterogeneous DGT devices; derivation of back percentage (*B*) for heterogeneous and homogeneous resin; flux diagram on how to determine *k*_d_ from *B* for heterogeneous and homogeneous models; determination of *k*_d_ from *B*; concentration profiles of Zn^+2^ and ZnL_Inorg_ for the heterogeneous resin model; derivation of maximum *B* in the homogeneous model; list of symbols; use of the excel file; and total concentrations introduced as an input in VMINTEQ (PDF)
