# Supplementary material for: Back Accumulation of Diffusive Gradients in Thin-Films Devices with a Stack of Resin Discs To Assess Availability of Metal Cations to Biota in Natural Waters
Source: Environ Sci Technol. 2023 May 15;57(20):7840–8. doi: 10.1021/acs.est.3c00799 (PMC10210536; doi:10.1021/acs.est.3c00799)
Supplement: Supplementary file 1 — es3c00799_si_001.pdf [file es3c00799_si_001.pdf]

Supporting Information for

Back accumulation of DGT (Diffusive Gradients in Thin-films) devices with a stack of resin discs to assess availability of metal cations to biota in natural waters

Jordi Sans-Duñó[1,2], Joan Cecilia[3], Josep Galceran[1], Jaume Puy[1]*, Willy Baeyens[2], Yue Gao [2]

1.Departament de Química. Universitat de Lleida, and AGROTECNIO-CERCA, Rovira Roure 191, 25198 Lleida, Catalonia, Spain

2. Analytical, Environmental & Geochemical (AMGC), Vrije Universiteit Brussel (VUB), Pleinlaan 2, B-1050 Brussels, Belgium

3. Departament de Matemàtica. Universitat de Lleida, and AGROTECNIO-CERCA, Rovira Roure 191, 25198 Lleida, Catalonia, Spain

*Corresponding author. E-mail address: jpuy@quimica.udl.cat, Phone 34 973 702529, Fax 34 973 702924

Contents

1 Homogeneous and heterogeneous resin models 3
2 Derivation of a solution for c_M and c_{ML} for a heterogeneous resin of a DGT device .. 6
3 Deriving the back percentage *B* for a heterogeneous resin 18
4 Deriving *B* for a homogeneous resin 19
5 Determination of k_d from *B* 22
5.1 Algorithm to obtain k_d using the complete expression for B corresponding to a homogeneous resins 22
5.2 Algorithm to obtain k_d for a heterogeneous resin 25
6 Concentration profiles of Zn^{+2} and pool of inorganic Zn complexes for an heterogeneous resin model 27
7 Derivation of maximum *B* for a homogeneous model 29
8 List of symbols 32
8.1 Latin symbols 32
8.2 Greek symbols 34
9 Use of Excel file to compute k_d with various models 34
9.1 How to enable Macros 34
9.2 Structure of the Excel file 34
10 Total concentrations introduced as input in VMINTEQ 37

1 Homogeneous and heterogeneous resin models

During the preparation of the Chelex resin discs, while the resin gels are becoming solid, the settling of the beads generates a heterogeneous distribution of the beads in the resin disc which concentrates the binding sites close to the bottom surface. We have used an optic microscope to show the heterogeneous distribution of the resin beads from the profile.

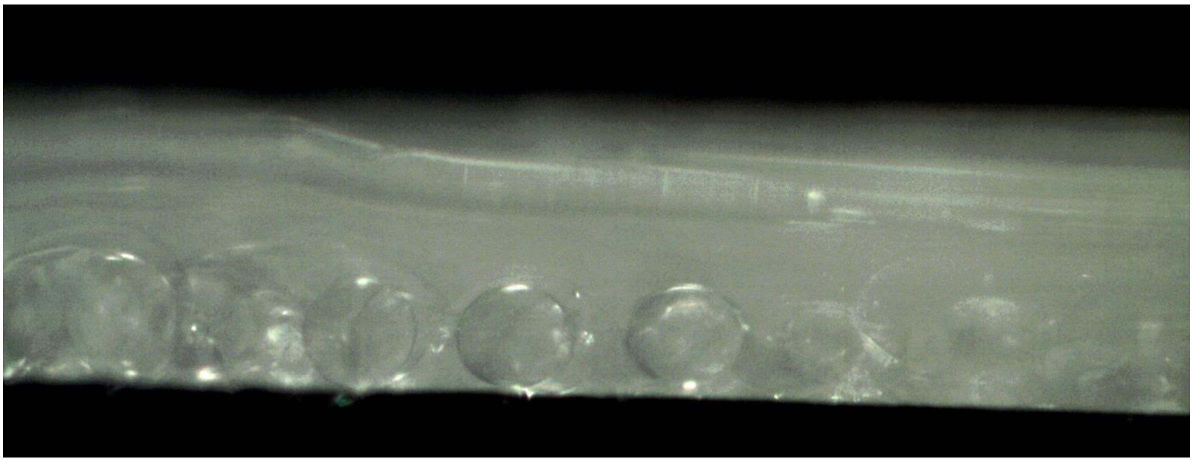

Figure S1 Photograph with an optic microscope of a Chelex resin. At the bottom of the picture we can see round shapes that correspond to Chelex 100 beads. On the bottom left of the picture there are different beads at different planes and that is why they are seen more blurred.

Therefore, it is convenient to develop a model that takes into account this heterogeneous distribution of the resin beads in the binding disc of a DGT device.

Let us consider a DGT device with a stack of two resin discs, whose resin beads have settled just in the front part of each resin disc (See Figure S2A). In order to assess for the relevance of the settling, we will use the homogeneous model as reference. In contrast to the heterogeneous model, in the homogeneous model, the binding sites are uniformly distributed within the whole resin disc (See Figure S2B).

Figure S2 is a schematic representation of the stack of the two resin discs. The x-axis is perpendicular to the interface between the resin disc and the diffuse disc. The resins are in the range $0<x<\delta^r$.

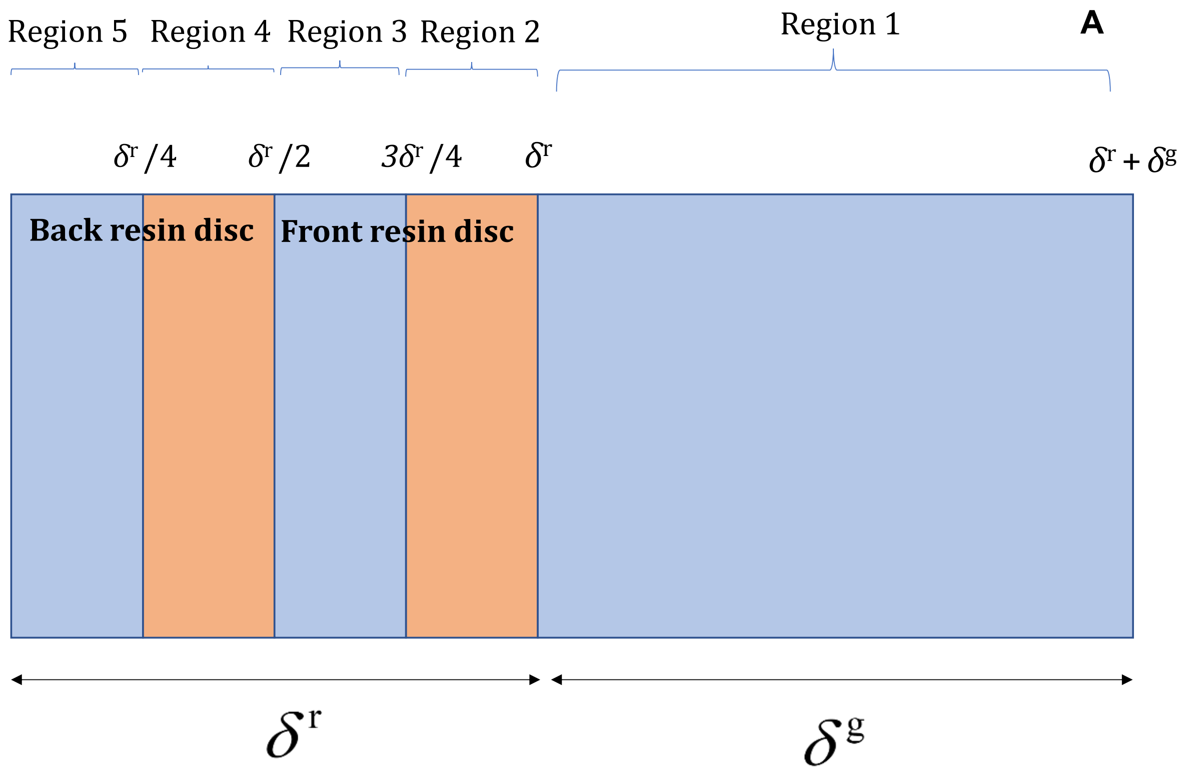

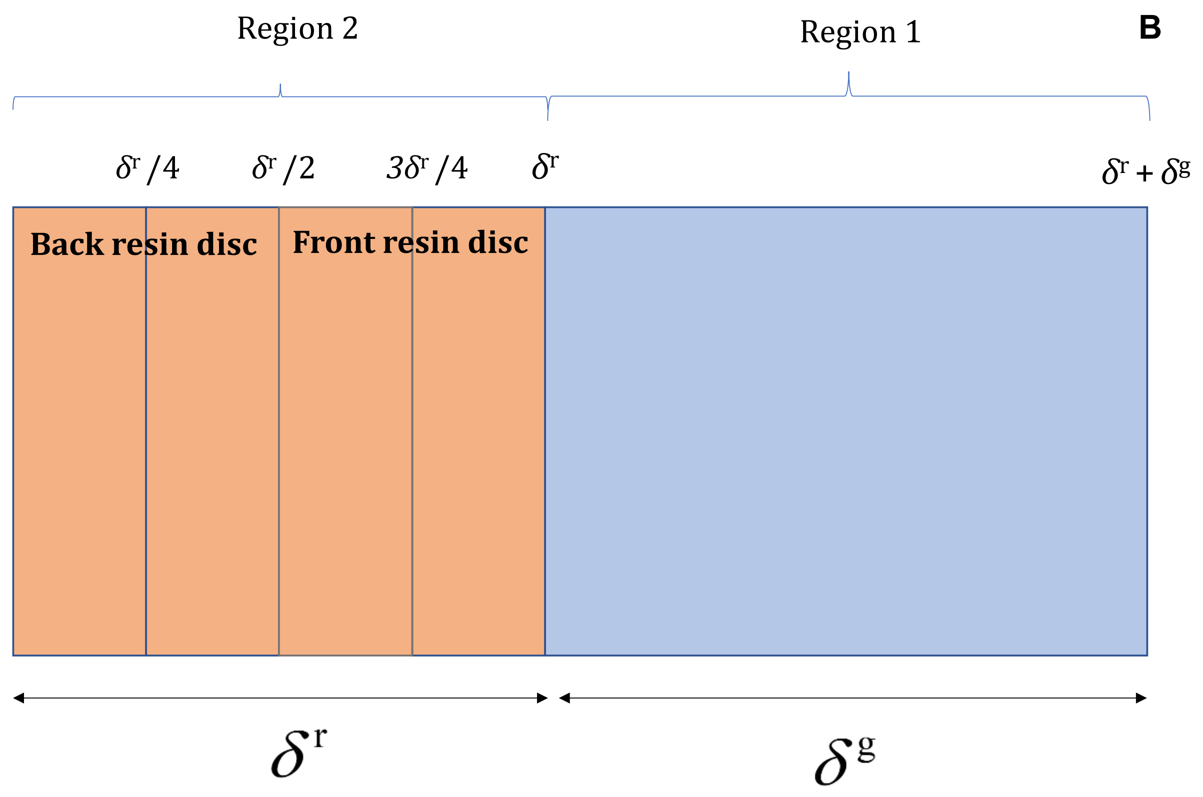

Figure S2 Domains considered in a heterogeneous distribution (panel A) and a homogeneous distribution of the resin beads (panel B) in a stack of two resin discs (front resin, $\delta^{r}/2<x<\delta^{r}$ and back resin $0<x<\delta^{r}/2$). Orange background indicates the presence of resin sites, while they are absent in the blue background shading. δ^{g} is the aggregate thickness of the diffusive disc plus that of the Diffusive Boundary Layer (DBL). δ^{r} is the aggregate thickness of the two resin discs.

In the next section, an analytical solution for the diffusion-reaction equations involving heterogeneous resin discs is reported.

2 Derivation of a solution for c_{M} and c_{ML} for a heterogeneous resin of a DGT device

We have developed a model that solves the diffusion-reaction equations for the boundary conditions of a DGT device considering a heterogeneous distribution of the beads of the resin in the resin discs.

Let us consider the simple case of a free metal (M) that reacts with a ligand (L) to produce a complex (ML).

$$\mathrm{M}+\mathrm{L} \underset{k_{\mathrm{d}}}{\overset{k_{\mathrm{a}}}{\rightleftharpoons}} \mathrm{ML}$$

The association reaction of M with L is considered a second order reaction rate with a constant (k_{a}) with units $\frac{\mathrm{m}^3}{\mathrm{mol\ s}}$ and the dissociation reaction is considered a first order rate with a constant (k_{d}) with units s^{-1}.

The equilibrium constant of this reaction process is defined as:

$$K=\frac{k_{\mathrm{a}}}{k_{\mathrm{d}}} \tag{S1}$$

We assume excess of ligand conditions, which imply that the concentration of L (c_{L}) is much larger than the concentration of M (c_{M}), so that it stays approximately constant everywhere in the DGT spatial domain. Therefore, a conditional equilibrium constant can be defined as:

$$K'=\frac{k_{\mathrm{a}}^{'}}{k_{\mathrm{d}}}=\frac{k_{\mathrm{a}}c_{\mathrm{L}}^{*}}{k_{\mathrm{d}}} \tag{S2}$$

Along the heterogeneous distribution presented in Figure S2A, the following assumptions are going to be considered to solve the diffusion-reaction equations:

- The resin domain is $\delta^{\mathrm{r}} > x > 0$, but only in the subdomains $\delta^{\mathrm{r}} \geq x \geq \frac{3\delta^{\mathrm{r}}}{4}$ and $\frac{\delta^{\mathrm{r}}}{2} \geq x \geq \frac{\delta^{\mathrm{r}}}{4}$ there is a constant concentration of resin beads (c_{R}^{*}). In the intervals $\frac{3\delta^{\mathrm{r}}}{4} \geq x \geq \frac{\delta^{\mathrm{r}}}{2}$ and $\frac{\delta^{\mathrm{r}}}{4} \geq x \geq 0$, c_{R}=0.
- The association of M with the resin beads is considered fast and strong, so perfect sink conditions for M are applied.
- The system is in steady-state conditions, so $\frac{\partial c_{\mathrm{M}}}{\partial t}=\frac{\partial c_{\mathrm{ML}}}{\partial t}=0$.

- In the bulk solution ($x>\delta^{r}+\delta^{g}$), the concentration of free metal (c_{M}) and the concentration of complex (c_{ML}) are considered constant ($c_{M} = c_{M}^{*}$ and $c_{ML} = c_{ML}^{*}$) and in equilibrium conditions, so $c_{ML}^{*} = K' c_{M}^{*}$.

Considering the previous assumptions and the schematic representation shown in Figure S2A , the steady state diffusion-reaction equations to be solved are

Mathematical formulation

Regions 1 $\left(\delta^{r} + \delta^{g} \geq x > \delta^{r}\right)$, **3** $\left(\frac{3\delta^{r}}{4} \geq x > \frac{\delta^{r}}{2}\right)$, **and 5** ($0 < x < \delta^{r}/4$):

$$D_{M} \frac{\partial^{2} c_{M}}{\partial x^{2}} + k_{d} c_{ML} - k_{a}^{'} c_{M} = 0 \qquad \text{(S3)}$$

$$D_{ML} \frac{\partial^{2} c_{ML}}{\partial x^{2}} - k_{d} c_{ML} + k_{a}^{'} c_{M} = 0 \qquad \text{(S4)}$$

Regions 2 ($\delta^{r} \geq x > \frac{3\delta^{r}}{4}$), **and 4** ($\frac{\delta^{r}}{2} \geq x > \frac{\delta^{r}}{4}$):

$$c_{M}(x)=0 \qquad \text{(S5)}$$

$$D_{ML} \frac{\partial^{2} c_{ML}}{\partial x^{2}} - k_{d} c_{ML} = 0 \qquad \text{(S6)}$$

Boundary conditions:

$$x = \delta^{r} + \delta^{g} \quad c_{M}(x = \delta^{r} + \delta^{g}) = c_{M}^{*}\,; \; c_{ML}(x = \delta^{r} + \delta^{g}) = c_{ML}^{*} \qquad \text{(S7)}$$

$$x = \delta^{r} \quad c_{M}\left(x = \delta^{r}\right) = 0 \;;\; c_{ML}(x = \delta^{r^{-}}) = c_{ML}(x = \delta^{r^{+}})\,;\; \left.\frac{\partial c_{ML}}{\partial x}\right|_{x=\delta^{r^{-}}} = \left.\frac{\partial c_{ML}}{\partial x}\right|_{x=\delta^{r^{+}}} \qquad \text{(S8)}$$

$$x = \frac{3\delta^{r}}{4} \quad c_{M}\left(x = \frac{3\delta^{r}}{4}\right) = 0 \;;\; c_{ML}(x = \frac{3\delta^{r^{-}}}{4}) = c_{ML}(x = \frac{3\delta^{r^{+}}}{4})\,;\; \left.\frac{\partial c_{ML}}{\partial x}\right|_{x=\frac{3\delta^{r^{-}}}{4}} = \left.\frac{\partial c_{ML}}{\partial x}\right|_{x=\frac{3\delta^{r^{+}}}{4}} \qquad \text{(S9)}$$

$$x = \frac{\delta^{r}}{2} \quad c_{M}\left(x = \frac{\delta^{r}}{2}\right) = 0 \;;\; c_{ML}(x = \frac{\delta^{r^{-}}}{2}) = c_{ML}(x = \frac{\delta^{r^{+}}}{2})\,;\; \left.\frac{\partial c_{ML}}{\partial x}\right|_{x=\frac{\delta^{r^{-}}}{2}} = \left.\frac{\partial c_{ML}}{\partial x}\right|_{x=\frac{\delta^{r^{+}}}{2}} \qquad \text{(S10)}$$

$$x = \frac{\delta^{r}}{4} \quad c_{M}\left(x = \frac{\delta^{r}}{4}\right) = 0 \;;\; c_{ML}(x = \frac{\delta^{r^{-}}}{4}) = c_{ML}(x = \frac{\delta^{r^{+}}}{4})\,;\; \left.\frac{\partial c_{ML}}{\partial x}\right|_{x=\frac{\delta^{r^{-}}}{4}} = \left.\frac{\partial c_{ML}}{\partial x}\right|_{x=\frac{\delta^{r^{+}}}{4}} \qquad \text{(S11)}$$

$$x = 0 \quad \left.\frac{\partial c_{M}}{\partial x}\right|_{x=0} = 0 \;;\; \left.\frac{\partial c_{ML}}{\partial x}\right|_{x=0} = 0 \qquad \text{(S12)}$$

Solution

In the gel layer (δ^{r} + $\delta^{\mathrm{g}} \geq x > \delta^{\mathrm{r}}$), which, according to the Figure S2A is referred as region 1, the transport equations are:

$$D_{\mathrm{M}} \frac{\partial^2 c_{\mathrm{M}}}{\partial x^2} + k_{\mathrm{d}} c_{\mathrm{ML}} - k_{\mathrm{a}}^{'} c_{\mathrm{M}} = 0 \qquad \text{(S13)}$$

$$D_{\mathrm{ML}} \frac{\partial^2 c_{\mathrm{ML}}}{\partial x^2} - k_{\mathrm{d}} c_{\mathrm{ML}} + k_{\mathrm{a}}^{'} c_{\mathrm{M}} = 0 \qquad \text{(S14)}$$

An equivalent system of equations that is uncoupled can be found by searching linear combinations of eqs (S13) and (S14). By addition, we obtain:

$$\frac{\partial^2 (D_{\mathrm{M}} c_{\mathrm{M}} + D_{\mathrm{ML}} c_{\mathrm{ML}})}{\partial x^2} = 0 \qquad \text{(S15)}$$

After solving a homogeneous second order differential equation, the general solution for eq (S15) is:

$$D_{\mathrm{M}} c_{\mathrm{M}} + D_{\mathrm{ML}} c_{\mathrm{ML}} = A_0 x + A_1 \qquad \text{(S16)}$$

A second independent uncoupled equation can be found for $\phi = c_{\mathrm{ML}} - K' c_{\mathrm{M}}$. Using eqs (S13) and (S14), after some rearrangements we obtain:

$$\frac{\partial^2 \phi}{\partial x^2} = \left(\frac{k_{\mathrm{a}}^{'}}{D_{\mathrm{M}}} + \frac{k_{\mathrm{d}}}{D_{\mathrm{ML}}} \right) \phi \qquad \text{(S17)}$$

The general solution for eq (S17) is

$$\phi = Q \exp\left(\frac{x}{m} \right) + P \exp\left(-\frac{x}{m} \right) \qquad \text{(S18)}$$

where $\frac{1}{m} = \sqrt{\frac{k_{\mathrm{a}}^{'}}{D_{\mathrm{M}}} + \frac{k_{\mathrm{d}}}{D_{\mathrm{ML}}}}$. Q and P can be redefined in terms of a new couple of constants *A*’ and *B*’ in order to obtain simplified expressions. In the case of the first region, these parameters are set equal to:

$$Q = \left(\frac{A' - B'}{2} \right) \exp\left(\frac{-(\delta^{\mathrm{r}} + \delta^{\mathrm{g}})}{m} \right) \qquad \text{(S19)}$$

$$P = \left(\frac{A' + B'}{2} \right) \exp\left(\frac{\delta^{\mathrm{r}} + \delta^{\mathrm{g}}}{m} \right) \qquad \text{(S20)}$$

Then ϕ in the first region has the following expression:

$$\phi = A'\cosh\left(\frac{\delta^{\mathrm{r}}+\delta^{\mathrm{g}}-x}{m}\right)+B'\sinh\left(\frac{\delta^{\mathrm{r}}+\delta^{\mathrm{g}}-x}{m}\right) \quad \text{(S21)}$$

Now, to obtain a particular solution for the free metal concentration and complex concentration, we need to apply the boundary conditions (from the first region) to find the particular solutions for eq (S16) and (S21). These conditions correspond to:

$$c_{\mathrm{M}}(x=\delta^{\mathrm{r}}+\delta^{\mathrm{g}})=c_{\mathrm{M}}^{*} \quad \text{(S22)}$$

$$c_{\mathrm{M}}\left(x=\delta^{\mathrm{r}}\right)=0 \quad \text{(S23)}$$

$$c_{\mathrm{ML}}(x=\delta^{\mathrm{r}}+\delta^{\mathrm{g}})=c_{\mathrm{ML}}^{*} \quad \text{(S24)}$$

$$c_{\mathrm{ML}}\left(x=\delta^{\mathrm{r}}\right)=c_{\mathrm{ML}}^{\mathrm{r}} \quad \text{(S25)}$$

where the superscript "r" indicates value of a concentration at $x=\delta^{\mathrm{r}}$. These values $\left(c_{\mathrm{M}}^{\mathrm{r}} \text{ and } c_{\mathrm{ML}}^{\mathrm{r}}\right)$, will be found later on (e.g. see eq (S63))

Once applied the boundary conditions (S22)-(S25), we obtain the solution for the integration constants:

$$A_0=\frac{D_{\mathrm{M}}c_{\mathrm{M}}^{*}+D_{\mathrm{ML}}(c_{\mathrm{ML}}^{*}-c_{\mathrm{ML}}^{\mathrm{r}})}{\delta^{\mathrm{g}}},\ A_1=D_{\mathrm{ML}}c_{\mathrm{ML}}^{\mathrm{r}}-\frac{D_{\mathrm{M}}c_{\mathrm{M}}^{*}+D_{\mathrm{ML}}(c_{\mathrm{ML}}^{*}-c_{\mathrm{ML}}^{\mathrm{r}})}{\delta^{\mathrm{g}}} \quad \text{(S26)}$$

and

$$A'=0,\ B'=\frac{c_{\mathrm{ML}}^{\mathrm{r}}}{\sinh\left(\frac{\delta^{\mathrm{g}}}{m}\right)} \quad \text{(S27)}$$

With the integration constants, we can obtain the concentration profiles in region 1 for c_{M} and c_{ML} as:

$$c_{\mathrm{M}}(x)=\frac{c_{\mathrm{M}}^{*}+\varepsilon(c_{\mathrm{ML}}^{*}-c_{\mathrm{ML}}^{\mathrm{r}})}{\delta^{\mathrm{g}}(1+\varepsilon K')}x-\frac{\varepsilon c_{\mathrm{ML}}^{\mathrm{r}}\sinh\left(\frac{\delta^{\mathrm{r}}+\delta^{\mathrm{g}}-x}{m}\right)}{(1+\varepsilon K')\sinh\left(\frac{\delta^{\mathrm{g}}}{m}\right)}+\frac{\delta^{\mathrm{g}}\varepsilon c_{\mathrm{ML}}^{\mathrm{r}}-(c_{\mathrm{M}}^{*}+\varepsilon(c_{\mathrm{ML}}^{*}-c_{\mathrm{ML}}^{\mathrm{r}}))\delta^{\mathrm{r}}}{\delta^{\mathrm{g}}(1+\varepsilon K')} \quad \text{(S28)}$$

and

$$c_{\mathrm{ML}}(x)=\frac{c_{\mathrm{ML}}^{*}+\varepsilon K'(c_{\mathrm{ML}}^{*}-c_{\mathrm{ML}}^{\mathrm{r}})}{\delta^{\mathrm{g}}(1+\varepsilon K')}x+\frac{c_{\mathrm{ML}}^{\mathrm{r}}\sinh\left(\frac{\delta^{\mathrm{r}}+\delta^{\mathrm{g}}-x}{m}\right)}{(1+\varepsilon K')\sinh\left(\frac{\delta^{\mathrm{g}}}{m}\right)}+\frac{\delta^{\mathrm{g}}\varepsilon K' c_{\mathrm{ML}}^{\mathrm{r}}-(c_{\mathrm{ML}}^{*}+\varepsilon K'(c_{\mathrm{ML}}^{*}-c_{\mathrm{ML}}^{\mathrm{r}}))\delta^{\mathrm{r}}}{\delta^{\mathrm{g}}(1+\varepsilon K')} \tag{S29}$$

where $\varepsilon=\frac{D_{\mathrm{ML}}}{D_{\mathrm{M}}}$, is the ratio of the diffusion coefficient of the metal complex and the free metal.

It is interesting to calculate $\left.\frac{\partial c_{\mathrm{ML}}}{\partial x}\right|_{x=\delta^{\mathrm{r}^-}}$ for the boundary condition involving the flux. From eq (S29), we obtain:

$$\left.\frac{\partial c_{\mathrm{ML}}}{\partial x}\right|_{x=\delta^{\mathrm{r}^+}}=\frac{c_{\mathrm{ML}}^{*}}{\delta^{\mathrm{g}}}-\frac{c_{\mathrm{ML}}^{\mathrm{r}}}{\delta^{\mathrm{g}}\left(1+\varepsilon K'\right)}\left(\varepsilon K'-\frac{\delta^{\mathrm{g}}\coth\left(\frac{\delta^{\mathrm{g}}}{m}\right)}{m}\right) \tag{S30}$$

This expression is of the interest to apply a boundary condition involving the flux at the right side of the resin-gel interface (eq (S30)) which will allow one to find an expression for $c_{\mathrm{ML}}^{\mathrm{r}}$ in terms of the physical parameters.

In region 2 ($\delta^{\mathrm{r}}\geq x\geq\frac{3\delta^{\mathrm{r}}}{4}$), c_{M}=0. The system of differential equations (S13)-(S14) is then reduced to:

$$D_{\mathrm{ML}}\frac{\partial^{2}c_{\mathrm{ML}}}{\partial x^{2}}-k_{\mathrm{d}}c_{\mathrm{ML}}=0 \tag{S31}$$

A general solution for eq (S31) is :

$$c_{\mathrm{ML}}(x)=A_{0}\sinh\left(\frac{\delta^{\mathrm{r}}-x}{\lambda_{\mathrm{ML}}}\right)+A_{1}\cosh\left(\frac{\delta^{\mathrm{r}}-x}{\lambda_{\mathrm{ML}}}\right) \tag{S32}$$

where $\lambda_{\mathrm{ML}}=\sqrt{\frac{D_{\mathrm{ML}}}{k_{\mathrm{d}}}}$.

Now, to obtain a particular solution for eq (S31), we need to apply the boundary conditions (S25) together with

$$c_{\mathrm{ML}}\left(x=\frac{3\delta^{\mathrm{r}}}{4}\right)=c_{\mathrm{ML}}^{\frac{3\mathrm{r}}{4}} \tag{S33}$$

where the superscript "3r/4" indicates that the value of the concentration is taken at $x=3\delta^{\mathrm{r}}/4$.

The solutions for the integration constants are:

$$A_0 = \frac{c_{\mathrm{ML}}^{\frac{3}{4}\mathrm{r}} - c_{\mathrm{ML}}^{\mathrm{r}} \cosh\left(\frac{\delta^{\mathrm{r}}}{4\lambda_{\mathrm{ML}}}\right)}{\sinh\left(\frac{\delta^{\mathrm{r}}}{4\lambda_{\mathrm{ML}}}\right)}, \quad A_1 = c_{\mathrm{ML}}^{\mathrm{r}} \tag{S34}$$

Substituting these integration constants, we obtain the concentration profile for the complex inside region 2, as a function of the boundary conditions and the physicochemical parameters.

$$c_{\mathrm{ML}}(x) = \frac{c_{\mathrm{ML}}^{\frac{3}{4}\mathrm{r}} - c_{\mathrm{ML}}^{\mathrm{r}} \cosh\left(\frac{\delta^{\mathrm{r}}}{4\lambda_{\mathrm{ML}}}\right)}{\sinh\left(\frac{\delta^{\mathrm{r}}}{4\lambda_{\mathrm{ML}}}\right)} \sinh\left(\frac{\delta^{\mathrm{r}} - x}{\lambda_{\mathrm{ML}}}\right) + c_{\mathrm{ML}}^{\mathrm{r}} \cosh\left(\frac{\delta^{\mathrm{r}} - x}{\lambda_{\mathrm{ML}}}\right) \tag{S35}$$

We can also calculate $\left.\frac{\partial c_{\mathrm{ML}}}{\partial x}\right|_{x=\delta^{\mathrm{r}^-}}$ and $\left.\frac{\partial c_{\mathrm{ML}}}{\partial x}\right|_{x=\frac{3\delta^{\mathrm{r}^-}}{4}}$, which will be useful to find an expression for $c_{\mathrm{ML}}^{\mathrm{r}}$ and $c_{\mathrm{ML}}^{\frac{3}{4}\mathrm{r}}$ in terms of the physicochemical parameters:

$$\left.\frac{\partial c_{\mathrm{ML}}}{\partial x}\right|_{x=\delta^{\mathrm{r}^-}} = \frac{c_{\mathrm{ML}}^{\mathrm{r}} \cosh\left(\frac{\delta^{\mathrm{r}}}{4\lambda_{\mathrm{ML}}}\right) - c_{\mathrm{ML}}^{\frac{3}{4}\mathrm{r}}}{\lambda_{\mathrm{ML}} \sinh\left(\frac{\delta^{\mathrm{r}}}{4\lambda_{\mathrm{ML}}}\right)} \tag{S36}$$

$$\left.\frac{\partial c_{\mathrm{ML}}}{\partial x}\right|_{x=\frac{3\delta^{\mathrm{r}^+}}{4}} = c_{\mathrm{ML}}^{\mathrm{r}} \left(\coth\left(\frac{\delta^{\mathrm{r}}}{4\lambda_{\mathrm{ML}}}\right)\cosh\left(\frac{\delta^{\mathrm{r}}}{4\lambda_{\mathrm{ML}}}\right) - \sinh\left(\frac{\delta^{\mathrm{r}}}{4\lambda_{\mathrm{ML}}}\right)\right) - \frac{c_{\mathrm{ML}}^{\frac{3}{4}\mathrm{r}} \coth\left(\frac{\delta^{\mathrm{r}}}{4\lambda_{\mathrm{ML}}}\right)}{\lambda_{\mathrm{ML}}} \tag{S37}$$

Let us, now, consider the region 3 ($\frac{3\delta^{\mathrm{r}}}{4} \geq x \geq \frac{\delta^{\mathrm{r}}}{2}$) where eq (S15) and eq (S14) have to be solved. The general solutions for c_{M} and c_{ML} are eqs (S16) and (S21).

In region 3, we have to apply eq (S33) and the following boundary conditions for the free metal and complex:

$$c_{\mathrm{M}}(x) = 0 \quad \text{for } x = \frac{3\delta^{\mathrm{r}}}{4} \text{ and } x = \frac{\delta^{\mathrm{r}}}{2} \tag{S38}$$

$$c_{\mathrm{ML}}\left(x=\frac{\delta^{\mathrm{r}}}{2}\right)=c_{\mathrm{ML}}^{\frac{\mathrm{r}}{2}} \quad \text{and} \quad c_{\mathrm{ML}}\left(x=\frac{3\delta^{\mathrm{r}}}{4}\right)=c_{\mathrm{ML}}^{\frac{3\mathrm{r}}{4}} \tag{S39}$$

The integration constants are:

$$A_0=\frac{4D_{\mathrm{ML}}\left(c_{\mathrm{ML}}^{\frac{3\mathrm{r}}{4}}-c_{\mathrm{ML}}^{\frac{\mathrm{r}}{2}}\right)}{\delta^{\mathrm{r}}},\ A_1=D_{\mathrm{ML}}c_{\mathrm{ML}}^{\frac{\mathrm{r}}{2}}+2D_{\mathrm{ML}}\left(c_{\mathrm{ML}}^{\frac{\mathrm{r}}{2}}-c_{\mathrm{ML}}^{\frac{3\mathrm{r}}{4}}\right) \tag{S40}$$

$$A'=\frac{c_{\mathrm{ML}}^{\frac{\mathrm{r}}{2}}-c_{\mathrm{ML}}^{\frac{3\mathrm{r}}{4}}\cosh\left(\frac{\delta^{\mathrm{r}}}{4m}\right)}{\sinh\left(\frac{\delta^{\mathrm{r}}}{4m}\right)},\ B'=c_{\mathrm{ML}}^{\frac{3\mathrm{r}}{4}} \tag{S41}$$

With the integration constants, we can write the concentration profiles in region 3 for c_{M} and c_{ML} in terms of the boundary values (e.g. $c_{\mathrm{ML}}^{\frac{\mathrm{r}}{2}}$) and the physicochemical parameters as:

$$c_{\mathrm{M}}(x)=\frac{\varepsilon\left(3c_{\mathrm{ML}}^{\frac{\mathrm{r}}{2}}-2c_{\mathrm{ML}}^{\frac{3\mathrm{r}}{4}}\right)}{(1+\varepsilon K')}+\frac{4\varepsilon\left(c_{\mathrm{ML}}^{\frac{3\mathrm{r}}{4}}-c_{\mathrm{ML}}^{\frac{\mathrm{r}}{2}}\right)}{\delta^{\mathrm{r}}(1+\varepsilon K')}x-\frac{c_{\mathrm{ML}}^{\frac{\mathrm{r}}{2}}-c_{\mathrm{ML}}^{\frac{3\mathrm{r}}{4}}\cosh\left(\frac{\delta^{\mathrm{r}}}{4m}\right)}{(1+\varepsilon K')\sinh\left(\frac{\delta^{\mathrm{r}}}{4m}\right)}\varepsilon\sinh\left(\frac{\frac{3}{4}\delta^{\mathrm{r}}-x}{m}\right)-\frac{\varepsilon c_{\mathrm{ML}}^{\frac{3\mathrm{r}}{4}}\cosh\left(\frac{\frac{3}{4}\delta^{\mathrm{r}}-x}{m}\right)}{(1+\varepsilon K')} \tag{S42}$$

and

$$c_{\mathrm{ML}}(x)=\frac{\varepsilon K'\left(3c_{\mathrm{ML}}^{\frac{\mathrm{r}}{2}}-2c_{\mathrm{ML}}^{\frac{3\mathrm{r}}{4}}\right)}{(1+\varepsilon K')}+\frac{4\varepsilon K'\left(c_{\mathrm{ML}}^{\frac{3\mathrm{r}}{4}}-c_{\mathrm{ML}}^{\frac{\mathrm{r}}{2}}\right)}{\delta^{\mathrm{r}}(1+\varepsilon K')}x+\frac{c_{\mathrm{ML}}^{\frac{\mathrm{r}}{2}}-c_{\mathrm{ML}}^{\frac{3\mathrm{r}}{4}}\cosh\left(\frac{\delta^{\mathrm{r}}}{4m}\right)}{(1+\varepsilon K')\sinh\left(\frac{\delta^{\mathrm{r}}}{4m}\right)}\sinh\left(\frac{\frac{3}{4}\delta^{\mathrm{r}}-x}{m}\right)+\frac{c_{\mathrm{ML}}^{\frac{3\mathrm{r}}{4}}\cosh\left(\frac{\frac{3}{4}\delta^{\mathrm{r}}-x}{m}\right)}{(1+\varepsilon K')} \tag{S43}$$

Now we can calculate $\left.\frac{\partial c_{\mathrm{ML}}}{\partial x}\right|_{x=\frac{3\delta^{\mathrm{r}-}}{4}}$ and $\left.\frac{\partial c_{\mathrm{ML}}}{\partial x}\right|_{x=\frac{\delta^{\mathrm{r}+}}{2}}$. We obtain:

$$\left.\frac{\partial c_{\mathrm{ML}}}{\partial x}\right|_{x=\frac{3\delta^{\mathrm{r}-}}{4}}=c_{\mathrm{ML}}^{\frac{3\mathrm{r}}{4}}\left(\frac{4\varepsilon K'}{\delta^{\mathrm{r}}\left(1+\varepsilon K'\right)}+\frac{\coth\left(\frac{\delta^{\mathrm{r}}}{4m}\right)}{m\left(1+\varepsilon K'\right)}\right)-c_{\mathrm{ML}}^{\frac{\mathrm{r}}{2}}\left(\frac{\operatorname{csch}\left(\frac{\delta^{\mathrm{r}}}{4m}\right)}{m\left(1+\varepsilon K'\right)}+\frac{4\varepsilon K'}{\delta^{\mathrm{r}}\left(1+\varepsilon K'\right)}\right) \tag{S44}$$

$$\left.\frac{\partial c_{\mathrm{ML}}}{\partial x}\right|_{x=\frac{\delta^{\mathrm{r}+}}{2}}=\frac{c_{\mathrm{ML}}^{\frac{3\mathrm{r}}{4}}\left(\frac{\cosh\left(\frac{\delta^{\mathrm{r}}}{4m}\right)\coth\left(\frac{\delta^{\mathrm{r}}}{4m}\right)-\sinh\left(\frac{\delta^{\mathrm{r}}}{4m}\right)}{m}+\frac{4\varepsilon K'}{\delta^{\mathrm{r}}}\right)}{\left(1+\varepsilon K'\right)}-\frac{c_{\mathrm{ML}}^{\frac{\mathrm{r}}{2}}\left(\frac{\coth\left(\frac{\delta^{\mathrm{r}}}{4m}\right)}{m}+\frac{4\varepsilon K'}{\delta^{\mathrm{r}}}\right)}{\left(1+\varepsilon K'\right)} \quad \text{(S45)}$$

From these expressions, later on, we are going to find $c_{\mathrm{ML}}^{\frac{3}{4}\mathrm{r}}$ and $c_{\mathrm{ML}}^{\frac{\mathrm{r}}{2}}$ in terms of the physicochemical parameters.

In region 4, ($\frac{\delta^{\mathrm{r}}}{2}\geq x\geq\frac{\delta^{\mathrm{r}}}{4}$), the same differential equation as in region 2 has to be solved, (eq (S31)) whose general solution is:

$$c_{\mathrm{ML}}(x)=A_0\sinh\left(\frac{\frac{\delta^{\mathrm{r}}}{2}-x}{\lambda_{\mathrm{ML}}}\right)+A_1\cosh\left(\frac{\frac{\delta^{\mathrm{r}}}{2}-x}{\lambda_{\mathrm{ML}}}\right) \quad \text{(S46)}$$

In this region 4, the boundary conditions to obtain the particular solution of the differential equation are (S39) together with

$$c_{\mathrm{ML}}\left(x=\frac{\delta^{\mathrm{r}}}{4}\right)=c_{\mathrm{ML}}^{\frac{\mathrm{r}}{4}} \quad \text{and} \quad c_{\mathrm{ML}}\left(x=\frac{\delta^{\mathrm{r}}}{2}\right)=c_{\mathrm{ML}}^{\frac{\mathrm{r}}{2}} \quad \text{(S47)}$$

The solutions for the integration constants are:

$$A_0=\frac{c_{\mathrm{ML}}^{\frac{\mathrm{r}}{4}}-c_{\mathrm{ML}}^{\frac{\mathrm{r}}{2}}\cosh\left(\frac{\delta^{\mathrm{r}}}{4\lambda_{\mathrm{ML}}}\right)}{\sinh\left(\frac{\delta^{\mathrm{r}}}{4\lambda_{\mathrm{ML}}}\right)},\ A_1=c_{\mathrm{ML}}^{\frac{\mathrm{r}}{2}} \quad \text{(S48)}$$

After obtaining the integration constants, the concentration profile for c_{ML} in region 4 becomes:

$$c_{\mathrm{ML}}(x)=\frac{c_{\mathrm{ML}}^{\frac{\mathrm{r}}{4}}-c_{\mathrm{ML}}^{\frac{\mathrm{r}}{2}}\cosh\left(\frac{\delta^{\mathrm{r}}}{4\lambda_{\mathrm{ML}}}\right)}{\sinh\left(\frac{\delta^{\mathrm{r}}}{4\lambda_{\mathrm{ML}}}\right)}\sinh\left(\frac{\delta^{\mathrm{r}}-x}{\lambda_{\mathrm{ML}}}\right)+c_{\mathrm{ML}}^{\frac{\mathrm{r}}{2}}\cosh\left(\frac{\delta^{\mathrm{r}}-x}{\lambda_{\mathrm{ML}}}\right) \quad \text{(S49)}$$

Now we can obtain $\left.\frac{\partial c_{\mathrm{ML}}}{\partial x}\right|_{x=\frac{\delta^{\mathrm{r}-}}{2}}$ and $\left.\frac{\partial c_{\mathrm{ML}}}{\partial x}\right|_{x=\frac{\delta^{\mathrm{r}+}}{4}}$ as:

$$\left.\frac{\partial c_{\mathrm{ML}}}{\partial x}\right|_{x=\frac{\vec{\delta}^{-}}{2}}=\frac{c_{\mathrm{ML}}^{\vec{2}}\cosh\left(\frac{\vec{\delta}}{4\lambda_{\mathrm{ML}}}\right)-c_{\mathrm{ML}}^{\vec{4}}}{\lambda_{\mathrm{ML}}\sinh\left(\frac{\vec{\delta}}{4\lambda_{\mathrm{ML}}}\right)} \quad \text{(S50)}$$

and

$$\left.\frac{\partial c_{\mathrm{ML}}}{\partial x}\right|_{x=\frac{\vec{\delta}^{+}}{4}}=\frac{\left(c_{\mathrm{ML}}^{\vec{2}}\cosh\left(\frac{\vec{\delta}}{4\lambda_{\mathrm{ML}}}\right)-c_{\mathrm{ML}}^{\vec{4}}\right)\cosh\left(\frac{\vec{\delta}}{4\lambda_{\mathrm{ML}}}\right)}{\lambda_{\mathrm{ML}}\sinh\left(\frac{\vec{\delta}}{4\lambda_{\mathrm{ML}}}\right)}-\frac{c_{\mathrm{ML}}^{\vec{2}}\sinh\left(\frac{\vec{\delta}}{4\lambda_{\mathrm{ML}}}\right)}{\lambda_{\mathrm{ML}}} \quad \text{(S51)}$$

From these expressions we will find an expression for $c_{\mathrm{ML}}^{\vec{2}}$ and $c_{\mathrm{ML}}^{\vec{4}}$ in terms of the physicochemical parameters.

In region 5, ($0 < x < \vec{\delta}/4$), the same system of uncoupled differential equations as in region 1 and 3 has to be solved (eqs (S15) and (S17). Their general solutions are eq (S16) and an equation similar to eq (S21) :

$$\phi=A'\cosh\left(\frac{\frac{1}{4}\vec{\delta}-x}{m}\right)+B'\sinh\left(\frac{\frac{1}{4}\vec{\delta}-x}{m}\right) \quad \text{(S52)}$$

Now to obtain a particular solution for the free metal concentration and complex concentration in region 5, we need to apply the boundary conditions.

$$c_{\mathrm{M}}\left(x=\frac{\vec{\delta}}{4}\right)=0 \quad \text{(S53)}$$

$$\left.\frac{\partial c_{\mathrm{M}}}{\partial x}\right|_{x=0}=0 \quad \text{(S54)}$$

$$c_{\mathrm{ML}}\left(x=\frac{\vec{\delta}}{4}\right)=c_{\mathrm{ML}}^{\vec{4}} \quad \text{(S55)}$$

$$\left.\frac{\partial c_{\mathrm{ML}}}{\partial x}\right|_{x=0}=0 \quad \text{(S56)}$$

The solutions for the integration constants are:

$$A_0=0, A_1=D_{\mathrm{ML}}c_{\mathrm{ML}}^{\vec{4}} \quad \text{(S57)}$$

$$A' = -c_{\mathrm{ML}}^{\frac{\mathrm{r}}{4}} \tanh\left(\frac{\delta^{\mathrm{r}}}{4m}\right), B' = c_{\mathrm{ML}}^{\frac{\mathrm{r}}{4}} \tag{S58}$$

With the integration constants, we obtain the concentration profiles for c_{ML} and c_{M} in region 5 as:

$$c_{\mathrm{M}}(x) = \frac{\varepsilon c_{\mathrm{ML}}^{\frac{\mathrm{r}}{4}}}{1+\varepsilon K'}\left(1+\tanh\left(\frac{\delta^{\mathrm{r}}}{4m}\right)\sinh\left(\frac{\frac{\delta^{\mathrm{r}}}{4}-x}{m}\right)-\cosh\left(\frac{\frac{\delta^{\mathrm{r}}}{4}-x}{m}\right)\right) \tag{S59}$$

and

$$c_{\mathrm{ML}}(x) = \frac{c_{\mathrm{ML}}^{\frac{\mathrm{r}}{4}}}{1+\varepsilon K'}\left(K'\varepsilon+\tanh\left(\frac{\delta^{\mathrm{r}}}{4m}\right)\sinh\left(\frac{\frac{\delta^{\mathrm{r}}}{4}-x}{m}\right)-\cosh\left(\frac{\frac{\delta^{\mathrm{r}}}{4}-x}{m}\right)\right) \tag{S60}$$

while $\left.\frac{\partial c_{\mathrm{ML}}}{\partial x}\right|_{x=\frac{\delta^{\mathrm{r}^+}}{4}}$ becomes:

$$\left.\frac{\partial c_{\mathrm{ML}}}{\partial x}\right|_{x=\frac{\delta^{\mathrm{r}^+}}{4}} = \frac{c_{\mathrm{ML}}^{\frac{\mathrm{r}}{4}} \tanh\left(\frac{\delta^{\mathrm{r}}}{4m}\right)}{m\left(1+\varepsilon K'\right)} \tag{S61}$$

Now that we have an expression for each region of our piecewise function, we can obtain the constants $c_{\mathrm{ML}}^{\mathrm{r}}, c_{\mathrm{ML}}^{\frac{3\mathrm{r}}{4}}, c_{\mathrm{ML}}^{\frac{\mathrm{r}}{2}}, c_{\mathrm{ML}}^{\frac{\mathrm{r}}{4}}$ in terms of the physical parameters by applying conditions of flux continuity.

In order to obtain $c_{\mathrm{ML}}^{\mathrm{r}}$, we can write:

$$D_{\mathrm{ML}} \left.\frac{\partial c_{\mathrm{ML}}}{\partial x}\right|_{x=\delta^{\mathrm{r}^+}} = D_{\mathrm{ML}} \left.\frac{\partial c_{\mathrm{ML}}}{\partial x}\right|_{x=\delta^{\mathrm{r}^-}} \tag{S62}$$

Using eqs (S30) and (S36) and solving for $c_{\mathrm{ML}}^{\mathrm{r}}$, a solution is found in terms of $c_{\mathrm{ML}}^{\frac{3\mathrm{r}}{4}}$ and some physical parameters:

$$c_{\mathrm{ML}}^{\mathrm{r}} = \frac{c_{\mathrm{ML}}^{*}(1+\varepsilon K')\lambda_{\mathrm{ML}} + c_{\mathrm{ML}}^{\frac{3\mathrm{r}}{4}}\delta^{\mathrm{g}}(1+\varepsilon K')\operatorname{csch}\left(\frac{\delta^{\mathrm{r}}}{4\lambda_{\mathrm{ML}}}\right)}{\varepsilon K'\lambda_{\mathrm{ML}} + \frac{\delta^{\mathrm{g}}\lambda_{\mathrm{ML}}}{m}\coth\left(\frac{\delta^{\mathrm{g}}}{m}\right) + \delta^{\mathrm{g}}(1+\varepsilon K')\coth\left(\frac{\delta^{\mathrm{r}}}{4\lambda_{\mathrm{ML}}}\right)} \tag{S63}$$

In order to obtain $c_{\mathrm{ML}}^{\frac{3\mathrm{r}}{4}}$, we apply:

$$D_{\mathrm{ML}} \left.\frac{\partial c_{\mathrm{ML}}}{\partial x}\right|_{x=\frac{3\delta^{\mathrm{r}^{+}}}{4}} = D_{\mathrm{ML}} \left.\frac{\partial c_{\mathrm{ML}}}{\partial x}\right|_{x=\frac{3\delta^{\mathrm{r}^{-}}}{4}} \tag{S64}$$

from which, using eqs (S37) and (S44) and solving for $c_{\mathrm{ML}}^{\frac{3\mathrm{r}}{4}}$, we find a solution in terms of $c_{\mathrm{ML}}^{\frac{\mathrm{r}}{2}}$ and some physical parameters. To write the solution for $c_{\mathrm{ML}}^{\frac{3\mathrm{r}}{4}}$ in a compact way, we define the variables *a, b* and *z* :

$$a = \left(\varepsilon K'\lambda_{\mathrm{ML}} + \frac{\delta^{\mathrm{g}}\lambda_{\mathrm{ML}}}{m}\coth\left(\frac{\delta^{\mathrm{g}}}{m}\right) + \delta^{\mathrm{g}}(1+\varepsilon K')\coth\left(\frac{\delta^{\mathrm{r}}}{4\lambda_{\mathrm{ML}}}\right)\right) \tag{S65}$$

$$b = a\left(\frac{4\varepsilon K'}{\delta^{\mathrm{r}}(1+\varepsilon K')} + \frac{\coth\left(\frac{\delta^{\mathrm{r}}}{4m}\right)}{m(1+\varepsilon K')} + \frac{\coth\left(\frac{\delta^{\mathrm{r}}}{4\lambda_{\mathrm{ML}}}\right)}{\lambda_{\mathrm{ML}}}\right) - \frac{\left(\left(\coth\left(\frac{\delta^{\mathrm{r}}}{4\lambda_{\mathrm{ML}}}\right)\cosh\left(\frac{\delta^{\mathrm{r}}}{4\lambda_{\mathrm{ML}}}\right) - \sinh\left(\frac{\delta^{\mathrm{r}}}{4\lambda_{\mathrm{ML}}}\right)\right)\operatorname{csch}\left(\frac{\delta^{\mathrm{r}}}{4\lambda_{\mathrm{ML}}}\right)\delta^{\mathrm{g}}(1+\varepsilon K')\right)}{\lambda_{\mathrm{ML}}} \tag{S66}$$

and

$$z = a\left(\frac{\operatorname{csch}\left(\frac{\delta^{\mathrm{r}}}{4m}\right)}{m(1+\varepsilon K')} + \frac{4\varepsilon K'}{\delta^{\mathrm{r}}(1+\varepsilon K')}\right) \tag{S67}$$

With these definitions,

$$c_{\mathrm{ML}}^{\frac{3\mathrm{r}}{4}} = \frac{c_{\mathrm{ML}}^{*}(1+\varepsilon K')\left(\coth\left(\frac{\delta^{\mathrm{r}}}{4\lambda_{\mathrm{ML}}}\right)\cosh\left(\frac{\delta^{\mathrm{r}}}{4\lambda_{\mathrm{ML}}}\right)-\sinh\left(\frac{\delta^{\mathrm{r}}}{4\lambda_{\mathrm{ML}}}\right)\right)+c_{\mathrm{ML}}^{\frac{\mathrm{r}}{2}}z}{b} \qquad \text{(S68)}$$

Then, to obtain $c_{\mathrm{ML}}^{\frac{\mathrm{r}}{2}}$, we apply:

$$D_{\mathrm{ML}}\left.\frac{\partial c_{\mathrm{ML}}}{\partial x}\right|_{x=\frac{\delta^{\mathrm{r}+}}{2}} = D_{\mathrm{ML}}\left.\frac{\partial c_{\mathrm{ML}}}{\partial x}\right|_{x=\frac{\delta^{\mathrm{r}-}}{2}} \qquad \text{(S69)}$$

which, using eqs (S45) and (S50) and solving for $c_{\mathrm{ML}}^{\frac{\mathrm{r}}{2}}$, has a solution in terms of $c_{\mathrm{ML}}^{\frac{\mathrm{r}}{4}}$ and some physical parameters. To be able to display the solution for $c_{\mathrm{ML}}^{\frac{\mathrm{r}}{2}}$, we use the previous variables b (eq (S66)), z (eq (S67)) and the following variables:

$$h = \frac{c_{\mathrm{ML}}^{*}(1+\varepsilon K')\left(\coth\left(\frac{\delta^{\mathrm{r}}}{4\lambda_{\mathrm{ML}}}\right)\cosh\left(\frac{\delta^{\mathrm{r}}}{4\lambda_{\mathrm{ML}}}\right)-\sinh\left(\frac{\delta^{\mathrm{r}}}{4\lambda_{\mathrm{ML}}}\right)\right)}{b}\left(\frac{\coth\left(\frac{\delta^{\mathrm{r}}}{4m}\right)\cosh\left(\frac{\delta^{\mathrm{r}}}{4m}\right)-\sinh\left(\frac{\delta^{\mathrm{r}}}{4m}\right)}{m(1+\varepsilon K')}+\frac{4\varepsilon K'}{\delta^{\mathrm{r}}(1+\varepsilon K')}\right) \qquad \text{(S70)}$$

and

$$f = \frac{-z}{b}\left(\frac{\coth\left(\frac{\delta^{\mathrm{r}}}{4m}\right)\cosh\left(\frac{\delta^{\mathrm{r}}}{4m}\right)-\sinh\left(\frac{\delta^{\mathrm{r}}}{4m}\right)}{m(1+\varepsilon K')}+\frac{4\varepsilon K'}{\delta^{\mathrm{r}}(1+\varepsilon K')}\right)+\frac{\coth\left(\frac{\delta^{\mathrm{r}}}{4\lambda_{\mathrm{ML}}}\right)}{\lambda_{\mathrm{ML}}}+\frac{\coth\left(\frac{\delta^{\mathrm{r}}}{4m}\right)}{m(1+\varepsilon K')}+\frac{4\varepsilon K'}{\delta^{\mathrm{r}}(1+\varepsilon K')} \qquad \text{(S71)}$$

With these definitions,

$$c_{\mathrm{ML}}^{\frac{\mathrm{r}}{2}} = \frac{c_{\mathrm{ML}}^{\frac{\mathrm{r}}{4}}\operatorname{csch}\left(\frac{\delta^{\mathrm{r}}}{4\lambda_{\mathrm{ML}}}\right)+h\lambda_{\mathrm{ML}}}{f\lambda_{\mathrm{ML}}} \qquad \text{(S72)}$$

Then, to obtain $c_{\mathrm{ML}}^{\frac{\mathrm{r}}{4}}$, we apply:

$$D_{\mathrm{ML}}\left.\frac{\partial c_{\mathrm{ML}}}{\partial x}\right|_{x=\frac{\delta^{\mathrm{r}+}}{4}} = D_{\mathrm{ML}}\left.\frac{\partial c_{\mathrm{ML}}}{\partial x}\right|_{x=\frac{\delta^{\mathrm{r}-}}{4}} \qquad \text{(S73)}$$

which, using eqs (S51) and (S61), has a solution only in terms of some physical parameters. To be able to show the solution for $\bar{c}_{\mathrm{ML}}^{4}$, we use the previous variables *f* (eq (S71)) and *h* (eq (S70)) and define the variable *p*:

$$p=\frac{\left(\coth\left(\frac{\delta^{\mathrm{r}}}{4\lambda_{\mathrm{ML}}}\right)\cosh\left(\frac{\delta^{\mathrm{r}}}{4\lambda_{\mathrm{ML}}}\right)-\sinh\left(\frac{\delta^{\mathrm{r}}}{4\lambda_{\mathrm{ML}}}\right)\right)}{f\lambda_{\mathrm{ML}}^{2}}\operatorname{csch}\left(\frac{\delta^{\mathrm{r}}}{4\lambda_{\mathrm{ML}}}\right)+\frac{\coth\left(\frac{\delta^{\mathrm{r}}}{4\lambda_{\mathrm{ML}}}\right)}{\lambda_{\mathrm{ML}}}+\frac{\tanh\left(\frac{\delta^{\mathrm{r}}}{4m}\right)}{m(1+\varepsilon K')} \tag{S74}$$

With these definitions,

$$\bar{c}_{\mathrm{ML}}^{4}=\frac{\left(\coth\left(\frac{\delta^{\mathrm{r}}}{4\lambda_{\mathrm{ML}}}\right)\cosh\left(\frac{\delta^{\mathrm{r}}}{4\lambda_{\mathrm{ML}}}\right)-\sinh\left(\frac{\delta^{\mathrm{r}}}{4\lambda_{\mathrm{ML}}}\right)\right)h}{fp\lambda_{\mathrm{ML}}} \tag{S75}$$

The ML concentration profile in the whole resin domain is a piecewise function defined by equations (S29), (S35), (S43) and (S49) which contain the boundary values given in eqns (S63), (S68), (S72) and (S75).

3 Deriving the back percentage *B* for a heterogeneous resin

To derive an expression to determine the percentage of back accumulation, *B* , one needs to calculate the ratio of accumulated moles of metal from *x*=0 to *x*=δ^{r}/2 over the accumulated moles of metal from *x*=0 to *x*= δ^{r} .

In order to derive this expression, first, one needs to add the differential equations for the free metal and complex (eq (S13) and eq (S14)). Second, one needs to integrate twice the resulting differential equation (S15). After obtaining the general solution (S16) and applying the boundary conditions (S22)-(S25), we obtain:

$$J\big|_{x=\delta^{\mathrm{r}}} = \frac{D_{\mathrm{M}} c_{\mathrm{M}}^{*}}{\delta^{\mathrm{g}}} + D_{\mathrm{ML}} \frac{c_{\mathrm{ML}}^{*} - c_{\mathrm{ML}}^{\mathrm{r}}}{\delta^{\mathrm{g}}} \qquad \text{(S76)}$$

A similar procedure leads to obtain the flux at $x = \delta^{\mathrm{r}/2}$, but one needs to apply the boundary conditions of the region 3 (eqs (S33), (S38) and (S39)).

$$J\big|_{x=\delta^{\frac{\mathrm{r}}{2}}} = 4D_{\mathrm{ML}} \left(\frac{c_{\mathrm{ML}}^{\frac{3\mathrm{r}}{4}} - c_{\mathrm{ML}}^{\frac{\mathrm{r}}{2}}}{\delta^{\mathrm{r}}} \right) \qquad \text{(S77)}$$

Then, to obtain *B* in a heterogeneous resin, one needs to divide the flux in the back resin (eq (S77)) by the total flux (eq (S76)):

$$B_{\mathrm{het}} = \frac{4D_{\mathrm{ML}} \left(\dfrac{c_{\mathrm{ML}}^{\frac{3\mathrm{r}}{4}} - c_{\mathrm{ML}}^{\frac{\mathrm{r}}{2}}}{\delta^{\mathrm{r}}} \right)}{\dfrac{D_{\mathrm{M}} c_{\mathrm{M}}^{*}}{\delta^{\mathrm{g}}} + \dfrac{D_{\mathrm{ML}} \left(c_{\mathrm{ML}}^{*} - c_{\mathrm{ML}}^{\mathrm{r}} \right)}{\delta^{\mathrm{g}}}} \qquad \text{(S78)}$$

4 Deriving *B* for a homogeneous resin

As above, to derive an expression for *B* in a homogenous resin, one needs to calculate the ratio of accumulated moles of metal from x=0 to $x=\delta^{\mathrm{r}}/2$ over the accumulated moles of metal from x=0 to $x=\delta^{\mathrm{r}}$.

In steady-state and ligand excess conditions, the accumulation when a metal (M) reacts with a ligand (L) to produce a complex (ML) can be written as:

$$n_{\mathrm{M}} = At\left(D_{\mathrm{ML}} \frac{\partial\, c_{\mathrm{ML}}}{\partial x}\bigg|_{x=\delta^{\mathrm{r}}} + D_{\mathrm{M}} \frac{\partial\, c_{\mathrm{M}}}{\partial x}\bigg|_{x=\delta^{\mathrm{r}}} \right) \tag{S79}$$

The flux is

$$J = D_{\mathrm{ML}} \frac{\partial\, c_{\mathrm{ML}}}{\partial x}\bigg|_{x=\delta^{\mathrm{r}}} + D_{\mathrm{M}} \frac{\partial\, c_{\mathrm{M}}}{\partial x}\bigg|_{x=\delta^{\mathrm{r}}} \tag{S80}$$

In the back resin (i.e $x<\delta^{\mathrm{r}}/2$) the accumulation is:

$$n_{\mathrm{M}} = At\, D_{\mathrm{M}} \frac{\partial c_{\mathrm{ML}}}{\partial x}\bigg|_{x=\frac{\delta^{\mathrm{r}}}{2}} \tag{S81}$$

With the same procedure used in the heterogenous resin, but using $c_{\mathrm{M}} = 0$ for the whole resin domain $0 < x < \delta^{\mathrm{r}}$, the flux at $x=\delta^{\mathrm{r}}$ can be written as,

$$J\big|_{x=\delta^{\mathrm{r}}} = \frac{D_{\mathrm{M}} c_{\mathrm{M}}^{*}}{\delta^{\mathrm{g}}} + D_{\mathrm{ML}} \frac{c_{\mathrm{ML}}^{*} - c_{\mathrm{ML}}^{\mathrm{r}}}{\delta^{\mathrm{g}}} \tag{S82}$$

The main difference in the flux calculation respect to the heterogeneous resin is found in the calculation of the flux of complex at $x=\delta^{\mathrm{r}}/2$.

Eq (S31) describes the diffusion-reaction equation for ML in a region with resin beads. This eq holds now for the complex in region 2 of the homogenous resin. The general solution of Eq (S31) can be written as:

$$c_{\mathrm{ML}}(x) = A_2 \exp\left(\frac{x}{\lambda_{\mathrm{ML}}}\right) + B_2 \exp\left(\frac{-x}{\lambda_{\mathrm{ML}}}\right) \tag{S83}$$

A particular solution can be found by applying the following boundary conditions:

$$c_{\mathrm{ML}}(x=\delta^{\mathrm{r-}}) = c_{\mathrm{ML}}^{\mathrm{r}} = c_{\mathrm{ML}}(x=\delta^{\mathrm{r+}})\,;\; \frac{\partial c_{\mathrm{ML}}}{\partial x}\bigg|_{x=\delta^{r-}} = \frac{\partial c_{\mathrm{ML}}}{\partial x}\bigg|_{x=\delta^{r+}} \tag{S84}$$

and

$$\left.\frac{\partial c_{\mathrm{ML}}}{\partial x}\right|_{x=0} = 0 \quad \text{(S85)}$$

The result for the integration constants A2 and B2 after applying the boundary conditions is :

$$A_2 = B_2 = \frac{c_{\mathrm{ML}}^{\mathrm{r}}}{2\cosh\left(\frac{\delta^{\mathrm{r}}}{\lambda_{\mathrm{ML}}}\right)}\ ;\ c_{\mathrm{ML}}^{\mathrm{r}} = c_{\mathrm{ML}}^{*}\left(\frac{\frac{\delta^{\mathrm{g}}}{m}\coth\left(\frac{\delta^{\mathrm{g}}}{m}\right)+\frac{\delta^{\mathrm{g}}(1+\varepsilon K')}{\lambda_{\mathrm{ML}}}\tanh\left(\frac{\delta^{\mathrm{r}}}{\lambda_{\mathrm{ML}}}\right)}{\varepsilon K'+\frac{\delta^{\mathrm{g}}}{m}\coth\left(\frac{\delta^{\mathrm{g}}}{m}\right)+\frac{\delta^{\mathrm{g}}(1+\varepsilon K')}{\lambda_{\mathrm{ML}}}\tanh\left(\frac{\delta^{\mathrm{r}}}{\lambda_{\mathrm{ML}}}\right)}\right) \quad \text{(S86)}$$

and the concentration profile after substituting the integration constants is:

$$c_{\mathrm{ML}}(x) = \frac{c_{\mathrm{ML}}^{\mathrm{r}}\cosh\left(\frac{x}{\lambda_{\mathrm{ML}}}\right)}{\cosh\left(\frac{\delta^{\mathrm{r}}}{\lambda_{\mathrm{ML}}}\right)} \quad \text{(S87)}$$

Applying $\left.\frac{\partial c_{\mathrm{ML}}}{\partial x}\right|_{x=\frac{\delta^{\mathrm{r}}}{2}}$ to the previous equation, the flux at $x=\delta^{\mathrm{r}}/2$ can be found as:

$$\left.J_{\mathrm{ML}}\right|_{x=\frac{\delta^{\mathrm{r}}}{2}} = D_{\mathrm{ML}}c_{\mathrm{ML}}^{\mathrm{r}}\frac{\sinh\left(\frac{\delta^{\mathrm{r}}}{2\lambda_{\mathrm{ML}}}\right)}{\lambda_{\mathrm{ML}}\cosh\left(\frac{\delta^{\mathrm{r}}}{\lambda_{\mathrm{ML}}}\right)} \quad \text{(S88)}$$

The expression for *B* in the homogeneous case results from dividing *J* at $x=\delta^{\mathrm{r}}$ (eq S82) by *J* at $x=\delta^{\mathrm{r}}/2$ (eq (S88)):

$$B = \frac{\varepsilon K'\,\mathrm{sech}\left(\frac{\delta^{\mathrm{r}}}{\lambda_{\mathrm{ML}}}\right)\sinh\left(\frac{\delta^{\mathrm{r}}}{2\lambda_{\mathrm{ML}}}\right)}{\lambda_{\mathrm{ML}}\coth\left(\frac{\delta^{\mathrm{g}}}{m}\right)+(1+\varepsilon K')m\tanh\left(\frac{\delta^{\mathrm{r}}}{\lambda_{\mathrm{ML}}}\right)} \quad \text{(S89)}$$

5 Determination of k_d from *B*

Sections 5.1 and 5.2 outline the determination of *B* from the limiting expression (8) of the main manuscript, from eq (S89) or from eq (S78). A short discussion on the validity conditions for using each one of these expression follows. Complementary to these Sections, an additional Excel file is included in the SI ready to estimate k_d value using to all these equations once input data from the user is introduced. The most accurate value of k_d appears in a green cell (See Section 9 in this file).

5.1 Algorithm to obtain k_d using the complete expression for B corresponding to a homogeneous resins

The use of the limiting expression eq (8) of the main manuscript, when applicable, allows a straightforward determination of k_d, while, when eq (S89) (eq (6) of the main manuscript) needs to be used, an iterative procedure can be applied.

In order to have a simple criterion for the plausibility of the application of eq (8), we explore the fulfilment of the approximation:

$$J_{\mathrm{M}}\Big|_{x=\delta^{\mathrm{r}}} << J_{\mathrm{ML}}\Big|_{x=\delta^{\mathrm{r}}} \tag{S90}$$

on which the limiting expression eq (8) relies. The reaction layer concept[30] applied to the DGT devices can provide approximate expressions for $J_{\mathrm{M}}\Big|_{x=\delta^{\mathrm{r}}}$ and $J_{\mathrm{ML}}\Big|_{x=\delta^{\mathrm{r}}}$.[23] Indeed, since almost all the metal arriving as free metal at $x=\delta^{\mathrm{r}}$ comes from the dissociation of complex in the reaction layer located inside the diffusive disc,

$$J_{\mathrm{M}}\Big|_{x=\delta^{\mathrm{r}}} \approx k_{\mathrm{d}} c_{\mathrm{ML}}^{*} \left(1-\xi\right) m \tanh\left(\frac{\delta^{\mathrm{g}}}{m}\right) \tag{S91}$$

where $c^{*}_{\mathrm{ML}}\left(1-\xi\right)$ stands for the complex concentration at the interface resin-diffusive disc, i.e., estimates the complex concentration taken as constant along the reaction layer inside the diffusive disc whose thickness is estimated with $m\tanh\left(\frac{\delta^{\mathrm{g}}}{m}\right)$. [17]

On the other hand, due to steady state, one can assume that the flux of arriving ML at $x=\delta^{\mathrm{r}}$ is also the amount of ML that is dissociating along an effective thickness of the complex penetration into the resin disc given by $\lambda_{\mathrm{ML}}\coth\left(\frac{\delta^{\mathrm{r}}}{\lambda_{\mathrm{ML}}}\right)$(see figure 1 in reference [17]) with average concentration $\frac{1}{2}c^{*}_{\mathrm{ML}}\left(1-\xi\right)$. Then,

$$J_{\mathrm{ML}}\Big|_{x=\delta^{\mathrm{r}}}\approx\frac{1}{2}k_{\mathrm{d}}c^{*}_{\mathrm{ML}}\left(1-\xi\right)\lambda_{\mathrm{ML}}\coth\left(\frac{\delta^{\mathrm{r}}}{\lambda_{\mathrm{ML}}}\right) \tag{S92}$$

According to eqs (S91) and (S92), the inequality $J_{\mathrm{M}}\Big|_{x=\delta^{\mathrm{r}}}<<J_{\mathrm{ML}}\Big|_{x=\delta^{\mathrm{r}}}$ reduces to $m\tanh\left(\frac{\delta^{\mathrm{g}}}{m}\right)<<\frac{1}{2}\lambda_{\mathrm{ML}}\coth\left(\frac{\delta^{\mathrm{r}}}{\lambda_{\mathrm{ML}}}\right)$ which considering the definitions of m and λ_{ML} given in eqs (4) and (3) of the main manuscript, can be written as

$$\sqrt{\frac{1}{1+\varepsilon K'}}\tanh\left(\frac{\delta^{\mathrm{g}}}{m}\right)\tanh\left(\frac{\delta^{\mathrm{r}}}{\lambda_{\mathrm{ML}}}\right)<<\frac{1}{2} \tag{S93}$$

as reported in the main manuscript.

The fulfillment of eq (S93) allows one to use the limiting expression (8) for the determination of k_{d}. Since both, m and λ_{ML} depend on k_{d}, we cannot verify eq (S93) until a provisional k_{d}-value was obtained from eq (8). The fulfilment of eq (S93) with this provisional value ensures that the k_{d} obtained is a good approximation for the dissociation rate constant of the complex. Conversely, if eq (S93) is not satisfied, a better approximation for k_{d} should be sought using eq (S89) instead of (8).

A simple, but effective, algorithm to calculate k_d using eq (S89) is explained as a flowchart in Figure S3.

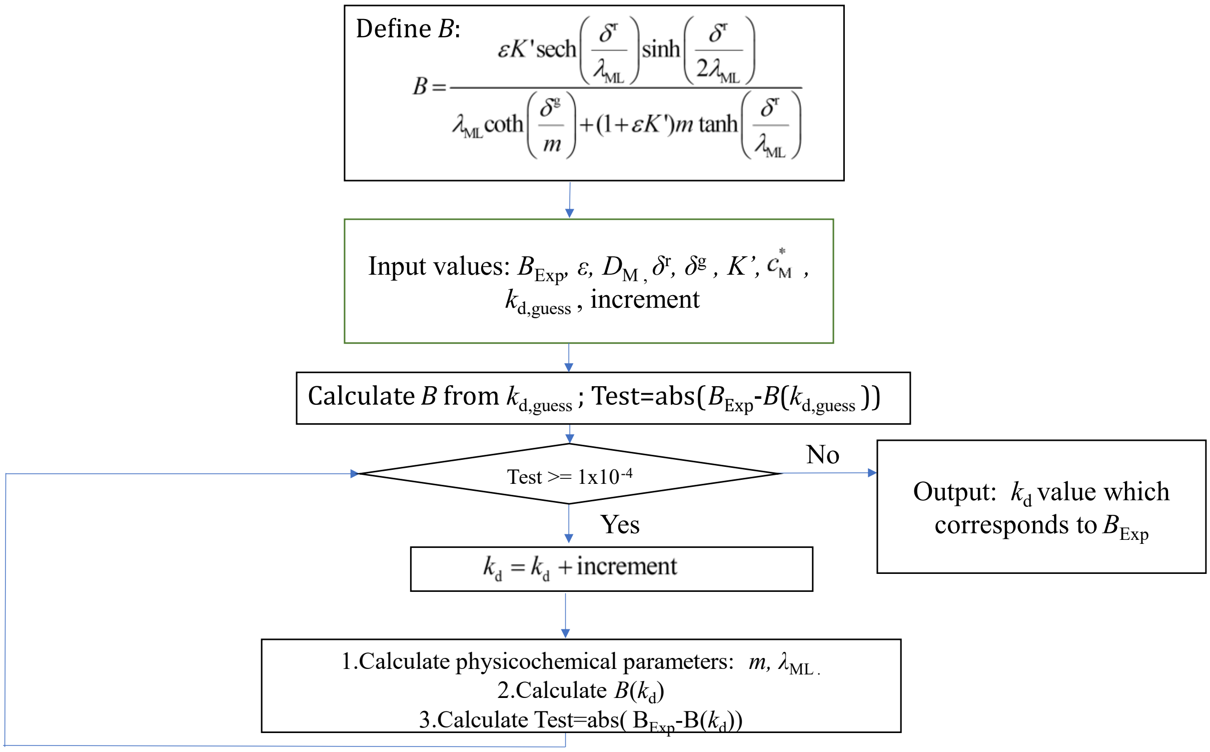

Figure S3 Algorithm applied to determine k_d from an experimental measurement of B_{exp} measured in Osor stream for a homogeneous resin.

The algorithm follows an iterative procedure that allows to obtain a value for k_d from an experimental B (in our work B_{Exp}=0.29 in the case of stream Osor). The algorithm searches the k_d result that satisfies the B_{exp} for a given tolerance (Test=1×10^{-4}) within a span of k_d values (1×10^{-1} s^{-1} to 1×10^{-6} s^{-1}). This iterative procedure needs an educated guess as an initial value for k_d which should be taken as a k_d=1×10^{-6} s^{-1} or k_d=1×10^{-1} s^{-1}. This educated guess can be assessed depending on the lability degree (ξ) of the complex. For ξ close to 0.7, k_d should be close to 1×10^{-2} s^{-1} (as it is expected for a quite labile complex), but for cases where ξ is close to 0.3, k_d will be close to 1×10^{-4} s^{-1} (as the behaviour of the complex should be quite inert). Therefore, in the first case a good educated guess would be 1×10^{-1} s^{-1} while for the second case a good educated guess would be 1×10^{-6} s^{-1}. Moreover, for the first case in this

algorithm it has to be chosen a negative increment (e.g increment=-1×10^{-6} s^{-1}), so the k_d value decreases and we search for a solution for partially labile complexes. While in the second case a positive increment is needed so the k_d increases and then we start searching for a solution of k_d for rather inert complexes. Solutions for k_d either for very labile or very inert complexes are not feasible with these method as transport is limited by diffusion and the kinetic reaction process does not have an impact on the accumulation.

Alternatively, a value for k_d can be obtained from high-level coding programs. For example, Mathematica has a subroutine called Findroot that helps to find a solution for a given equation or system of equations.

With the solution, we need to check whether the heterogeneity of the resin beads distribution is relevant for the determination of k_d. To do so, we need to check that $B_{exp}<B_{max}$, where B_{max} is the maximum back percentage that can be reached in a stack of two homogeneous resin discs. When B_{exp} is close to B_{max}, an accurate computation of k_d requires the use of the heterogeneous resin model, by means of eq (S78), as outlined in the following Section.

5.2 Algorithm to obtain k_d for a heterogeneous resin

According to fig (2) in the main manuscript, eq (S89) for the homogeneous model plotted against $k_{d,}$ has a maximum, that can be labelled as B_{max} . B_{max} is then, the maximum back percentage that can be reached by a stack of two homogeneous resin discs. Section "Derivation of maximum *B* for a homogeneous model" gives some details on the calculation of B_{max}.

When B_{exp} is close or greater than B_{max}, eq (S78) is needed to recover accurate k_d values. In this task, we can use essentially the same algorithm as in section "Derivation of a solution for c_M and c_{ML} for a heterogeneous resin of a DGT device ".

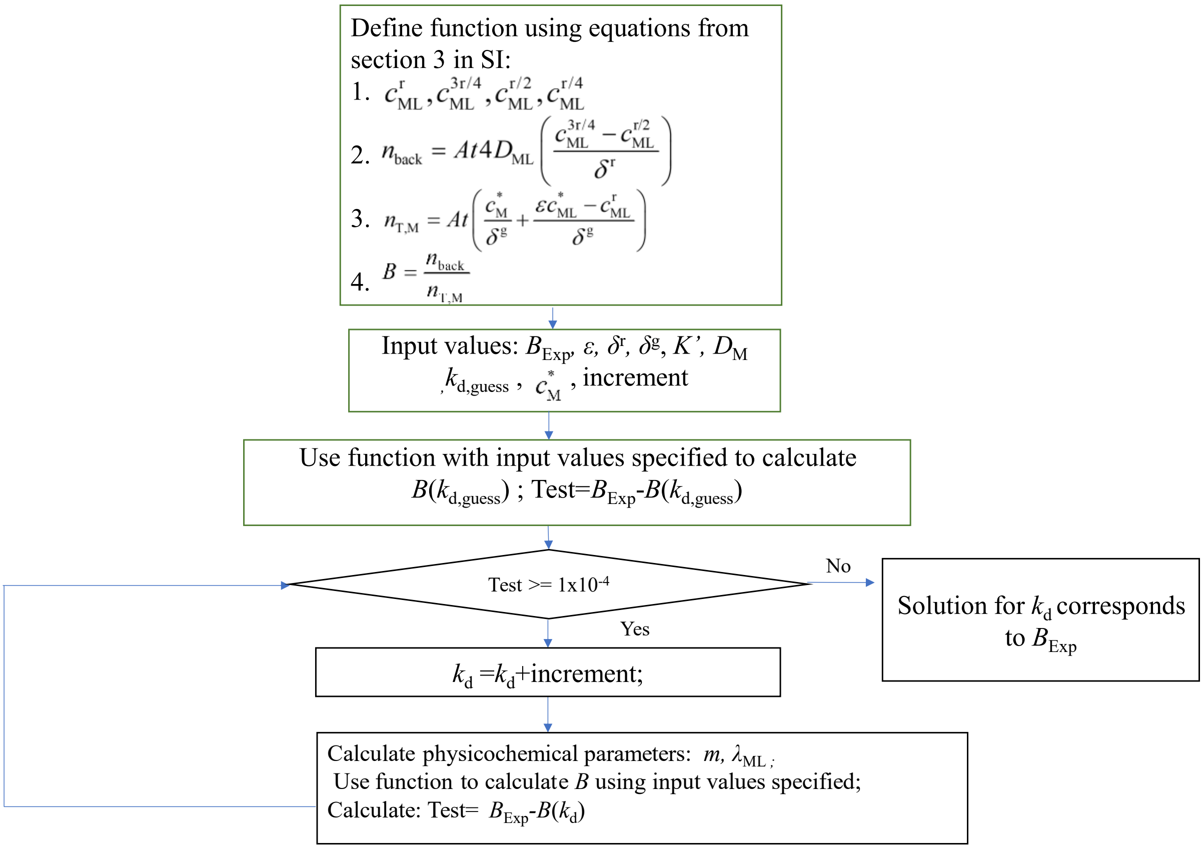

Figure S4 Algorithm applied to determine k_{d} from a B measured in Osor stream for a heterogeneous resin.

In the flowchart shown in Figure S4, $c_{\mathrm{ML}}^{\mathrm{r}}$, $c_{\mathrm{ML}}^{3\mathrm{r}/4}$ and $c_{\mathrm{ML}}^{\mathrm{r}/2}$ and $c_{\mathrm{ML}}^{\mathrm{r}/4}$ can be computed with eqs (S63), (S68), (S72) and (S75), respectively.

Once they are written down in the program, B can be computed as prescribed in eq (S78). Once again, notice that this procedure is the algorithm that has been used in this work, nevertheless other programs can be applied to iteratively solve the equation and find a solution for k_{d} that satisfies the B_{exp}.

6 Concentration profiles of Zn^{+2} and pool of inorganic Zn complexes for an heterogeneous resin model

Using the equations derived in section "Derivation of a solution for c_M and c_{ML} for a heterogeneous resin of a DGT device " and the data from Osor stream (regarding equilibrium concentrations, kinetic dissociation constant and diffusion coefficients), one can compute the concentration profiles for the free metal and the complex with the boundary conditions of the DGT device equipped with a stack of two resin discs. For the standard thickness of the resin disc, 0.4 mm, the back disc occupies the abscissas axis in the range $0< x< 0.4$ mm, while the front resin disc is located in the abscissa range 0.4 mm $< x <$0.8 mm.

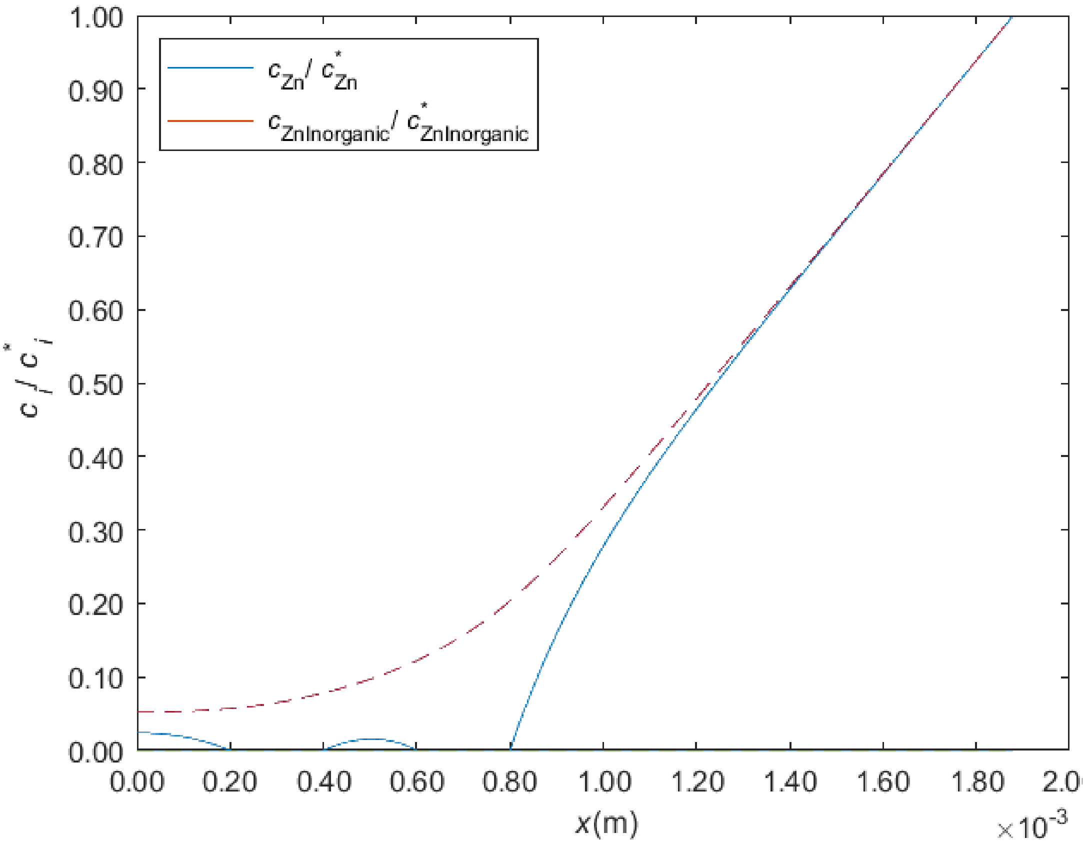

Figure S5 Normalized concentration profiles in a stack of 2 heterogeneous resin discs.The continuous blue line shows the concentration profile for free zinc, the dashed red line shows the concentration profile for a pool of inorganic complexes ZnL_{Inor}.The thickness of the resin layer and gel layer are $\delta^{r}=8\times10^{-4}$ m and $\delta^{g}=1.079\times10^{-4}$ m, respectively. The diffusion coefficients of complex and free metal are $D_{ZnLinor}=D_{Zn}=4.21\times10^{-10}$ m^2 s^{-1}. The kinetic dissociation constant is $k_d=3.35\times10^{-3}$ s^{-1} and the conditional stability constant is $K'=4.70$ mol m^{-3}.

Figure S5 depicts the concentration profiles of free metal and complex in a stack of two heterogeneous resin discs (as illustrated in Figure S2). This figure is quite useful to understand that the percentage of back accumulation, when using heterogeneous resin discs, is higher than the back accumulation when the resin discs are homogeneous. Dissociation of the complex in the whole volume $0 < x < 0.4$ mm leads to back accumulation when the resins are homogeneous. Instead, dissociation in the volume $0 < x < 0.5$ mm leads to back accumulation when the resins

are heterogeneous. Notice in Figure S5 that in the volume $0<x<0.2$ mm where there is no resin beads, a profile of free metal resulting from the complex dissociation appears. The decreasing c_{M} concentration as x increases indicates that the free metal produced in this volume diffuses towards increasing x values and gets bound to the back resin at x=0.2 mm. Likewise, a free metal concentration appears in the volume 0.4 mm < x <0.6 mm. The profile of the free metal (continuous blue line in Figure S5) indicates that the metal produced between 0.4 mm < x< 0.5 mm diffuses also towards the back resin disc and binds to it (at x=0.4 mm) increasing the back accumulation with respect to what takes place when the resin discs are homogeneous.

7 Derivation of maximum *B* for a homogeneous model

Back accumulation in a DGT device is negligible for a fully labile system as well as for a completely inert one. In both cases there is no dissociation of complexes in the resin domain, which is the phenomenon responsible for the back accumulation assuming that the resin beads are homogeneously distributed in the resin disc with a concentration enough to bind all the metals that diffuse in this domain (i.e. no saturation effects).

The accumulation in the back resin disc depends on the physicochemical parameters of the metal species (D_{M}, D_{ML}, K', k_{d}), as well on the characteristics of a DGT device (δ^{g} and δ^{r}). In this Section, we are going to derive a condition to determine the dissociation rate constant of the complex that yields to the maximum percentage of back accumulation, assuming fixed values of the rest of parameters.

The partial derivative of B respect to k_{d} in eq (S89) is

$$\frac{\partial B}{\partial k_{\mathrm{d}}}=\frac{\lambda_{\mathrm{ML}}K'\operatorname{sech}\left(\frac{\delta^{\mathrm{r}}}{\lambda_{\mathrm{ML}}}\right)\left(\delta^{\mathrm{r}}\cosh\left(\frac{\delta^{\mathrm{r}}}{2\lambda_{\mathrm{ML}}}\right)\alpha+2\sinh\left(\frac{\delta^{\mathrm{r}}}{2\lambda_{\mathrm{ML}}}\right)\left(\left(\delta^{\mathrm{g}}\sqrt{1+\varepsilon K'}\right)\operatorname{csch}\left(\frac{\delta^{\mathrm{g}}\sqrt{1+\varepsilon K'}}{\lambda_{\mathrm{ML}}}\right)^{2}-\delta^{\mathrm{r}}\beta\right)\right)}{4D_{\mathrm{M}}\sqrt{1+\varepsilon K'}\alpha^{2}} \quad \text{(S94)}$$

where $\lambda_{\rm ML} = \sqrt{\frac{D_{\rm ML}}{k_{\rm d}}}$ and

$$\alpha = \coth\left(\frac{\delta^{\rm g}\sqrt{1+\varepsilon K'}}{\lambda_{\rm ML}}\right) + \sqrt{1+\varepsilon K'}\tanh\left(\frac{\delta^{r}}{\lambda_{\rm ML}}\right) \quad \text{(S95)}$$

and

$$\beta = \sqrt{1+\varepsilon K'} + \coth\left(\frac{\delta^{\rm g}\sqrt{1+\varepsilon K'}}{\lambda_{\rm ML}}\right)\tanh\left(\frac{\delta^{r}}{\lambda_{\rm ML}}\right) \quad \text{(S96)}$$

The condition of maximum requires that this derivative is equal 0. Unfortunately, there is not an explicit solution of equation (S94) for $k_{\rm d}$. Therefore, the Bolzano method has been applied to find the $k_{\rm d}$ value ($k_{\rm d,max}$) which produces a 0 in eq (S94), where $B(k_{\rm d,max}) = B_{\rm max}$ .The initial points that define the starting section are selected as $k_{\rm d,a}=0.1$ s^{-1} and $k_{\rm d,b}=1\times10^{-9}$ s^{-1} . To calculate a middle point $k_{\rm d,c}$ between $k_{\rm d,a}$ and $k_{\rm d,b}$, we have used $\log(k_{\rm d,c})=(\log(k_{\rm d,a})+\log(k_{\rm d,b}))/2$. The solution for $k_{\rm d}$ is accepted once the condition $(k_{\rm d,a}-k_{\rm d,b})/2<$tolerance is satisfied, where tolerance=1×10^{-9} s^{-1}.

The Bolzano method can be applied iteratively for a range of K' values, so that we can plot a figure of $B_{\rm max}$ vs K'. We have computed this relationship for the following parameters $\delta^{\rm g}=1.079\times10^{-3}$ m, $\delta^{\rm r}=8\times10^{-4}$ m, $D_{\rm ML}=D_{\rm M}=4.20\times10^{-10}$ m^2 s^{-1}and the result is shown in Figure S6 .

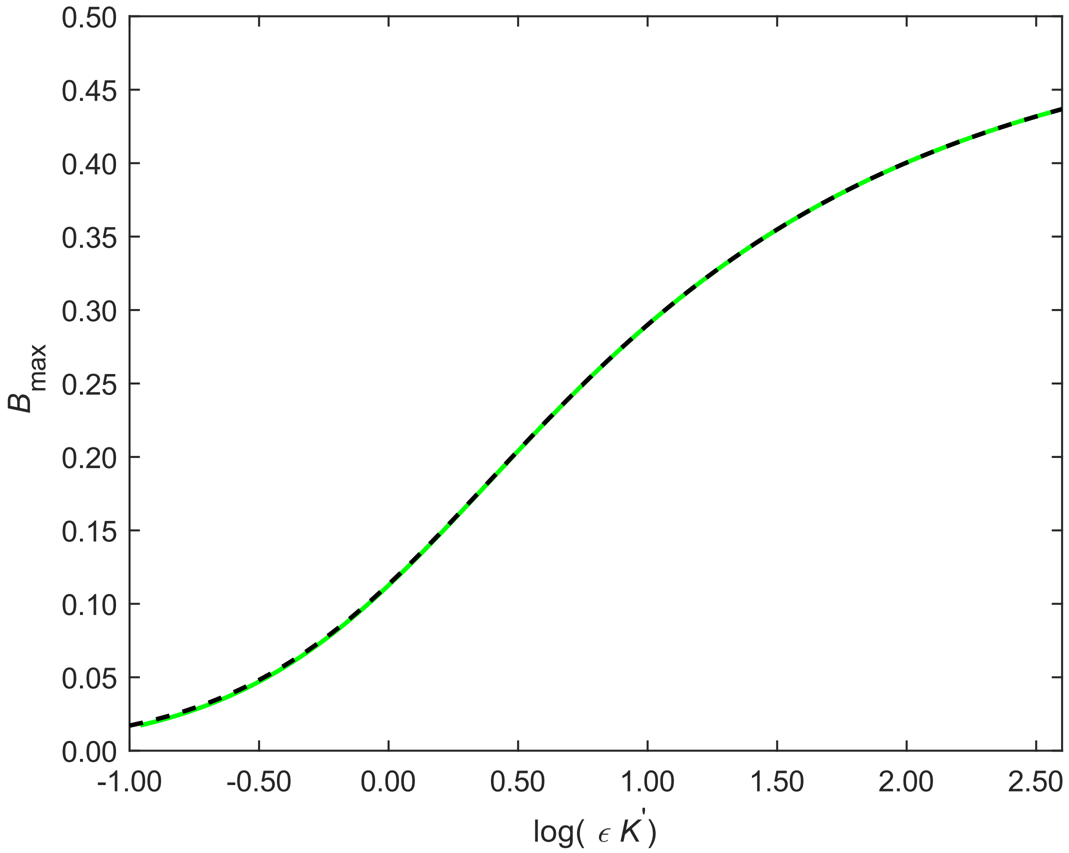

Figure S6 : Impact of $\varepsilon K'$ on $B_{max,}$ the dashed black line shows the behaviour of B_{max} (computed with the homogeneous model) against log ($\varepsilon K'$) for the parameters of δ^{g} =1.079x10^{-3} m, δ^{r} =8x10^{-4} m, D_{M}=4.20x10^{-10} m s^{-2} and ε=0.1. The solid green line is calculated with the same parameters as the dashed black line but using ε=1.

This figure is of high interest to assess whether the homogeneous model of the resin disc is accurate for determining k_{d} or the heterogeneous model of the resin disc is required. As seen in Fig. 1 of the main text, when the percentage of back accumulation (B_{exp}) approaches the maximum value of the homogeneous model (B_{max}), an accurate value of k_{d} will require the use of eq (S78). Figure S6 allows one to check whether B_{exp} is close to the maximum B (B_{max}) for a given K' and standard δ^{g} =1.079×10^{-3} m and δ^{r} =8×10^{-4} m values. Figure S6 also shows that a change of ε from 1 (solid green line) to 0.1 (dashed black line) leads to just small

discrepancies in B_{max}, when $\varepsilon K'$ is smaller than 1. Thus, this plot can be used for the typical values in standard DGT devices.

Just as an example, we can consider the case of Zn in the Osor stream. We have K'=4.70 and ε=1, so log($\varepsilon K'$)=0.67. From Figure S6 , we know that the maximum value that can be achieved for B is near to 0.22. As in our case we have B_{exp}=0.29 , it is not possible to find a solution for k_d using the homogeneous model. Then, instead of applying eq (S89) we will need to apply eq (S78). In the case that $B_{exp}<B_{max}$, then we can find a solution for k_d applying the homogeneous model (eq(S89)) and from our computations we know -for these data- that the k_d retrieved by the heterogeneous model will be just 0.3 log units greater than the result retrieved by the homogeneous model which corresponds to a k_d value 2 times greater.

8 List of symbols

8.1 Latin symbols

A : Area

B: theoretical value *%back* homogeneous

B_{exp}: Experimental value *%back*

B_{het}: theoretical value *%back* heterogeneous model

B_{max}: maximum value *%back* for homogeneous model

c_L^*: Concentration of the ligand in the bulk solution

c_M^* : Concentration of free metal ion in the bulk solution

c_{ML}^*: Concentration of the complex in the bulk solution

c_{ML}^{r}: concentration of complex at $x = \delta^{r}$

$c_{ML}^{3r/4}$: concentration of complex at $x=3\delta^{r}/4$

$c_{ML}^{r/2}$: concentration of complex at $x= \delta^{r}/2$

$c_{ML}^{r/4}$: concentration of complex at $x= \delta^{r}/4$

c_{R} : concentration of resin beads

D_{M} : diffusion coefficient of the free metal ion

D_{ML} : diffusion coefficient of the complex

J: total metal flux at the resin-gel interface

J_{ML}: Flux of complex at the resin interface

J_{M}: Flux of free metal at the resin interface

K: Equilibrium constant

K': Conditional equilibrium constant

k_{a}: association rate constant of the complex

k_{d}: dissociation rate constant of the complex

L: Ligand

M: free metal ion

m: reaction layer thickness

ML: complex

n_{M} : total metal accumulation at the resin interface

t: time

8.2 Greek symbols

δ^{g}: Thickness diffusive gel layer + thickness of DBL

δ^{r}: Thickness resin layer

ε: ratio of the diffusion coefficient of the complex divided by the diffusion coefficient of the free metal ion

λ_{ML}: penetration distance of the complex in the resin layer

ξ: lability degree

9 Use of Excel file to compute k_d with various models

9.1 How to enable Macros

The use of the Excel file requires that Macros were enabled. Usually Microsoft displays a yellow warning which reads: “Security Warning: Macros have been disabled”. This message comes with a button that enables the Macros in your excel file.

If this message does not appear in your screen, please look at internet for how to enable macros for Excel taking into account your Excel version and your operative system.

9.2 Structure of the Excel file

The Excel file contains 3 sheets: “Input”, “Intermediary calculations” and “Output”.

In the Input sheet, you can find the list of Input values that are needed to calculate “k_d”. By default, the list of entries is filled with the values from the measurements in natural waters in the Osor stream:

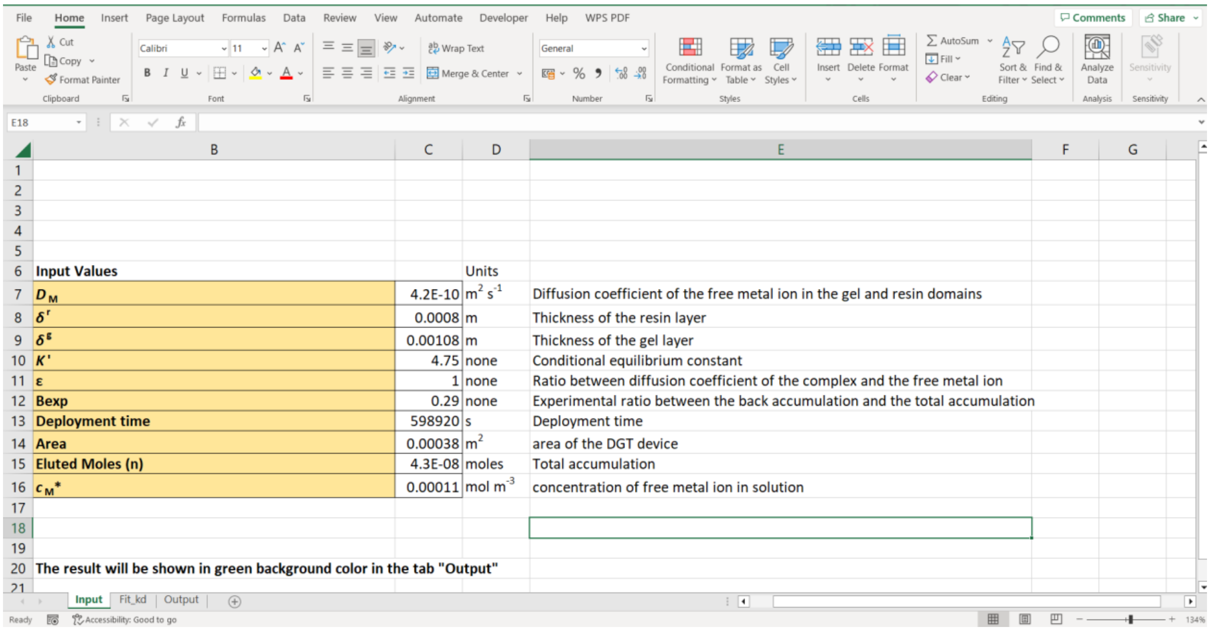

	B	C	D	E
6	**Input Values**		Units	
7	**D_M**	4.2E-10	m^2 s^{-1}	Diffusion coefficient of the free metal ion in the gel and resin domains
8	**δ^r**	0.0008	m	Thickness of the resin layer
9	**δ^g**	0.00108	m	Thickness of the gel layer
10	**K'**	4.75	none	Conditional equilibrium constant
11	**ε**	1	none	Ratio between diffusion coefficient of the complex and the free metal ion
12	**Bexp**	0.29	none	Experimental ratio between the back accumulation and the total accumulation
13	**Deployment time**	598920	s	Deployment time
14	**Area**	0.00038	m^2	area of the DGT device
15	**Eluted Moles (n)**	4.3E-08	moles	Total accumulation
16	**c_M***	0.00011	mol m^{-3}	concentration of free metal ion in solution
20	**The result will be shown in green background color in the tab "Output"**			

Figure S7 List of Input values from the measurements in Osor stream with the units used by the program to compute k_d (s^{-1}).

Figure S7 displays the list of Input values to be provided by the user in order to fit a k_d value from B_{exp}.

The second sheet (Figure S8), named “Intermediary calculations”, is used for the automatic computation of k_d according to the homogeneous resin model, when $B_{exp} < B_{max}$, or to the heterogeneous model when $B_{exp} > B_{max}$. As 2 solutions can be obtained, the excel file uses the accumulation in the DGT device to select the correct solution. The user does not need to change anything.

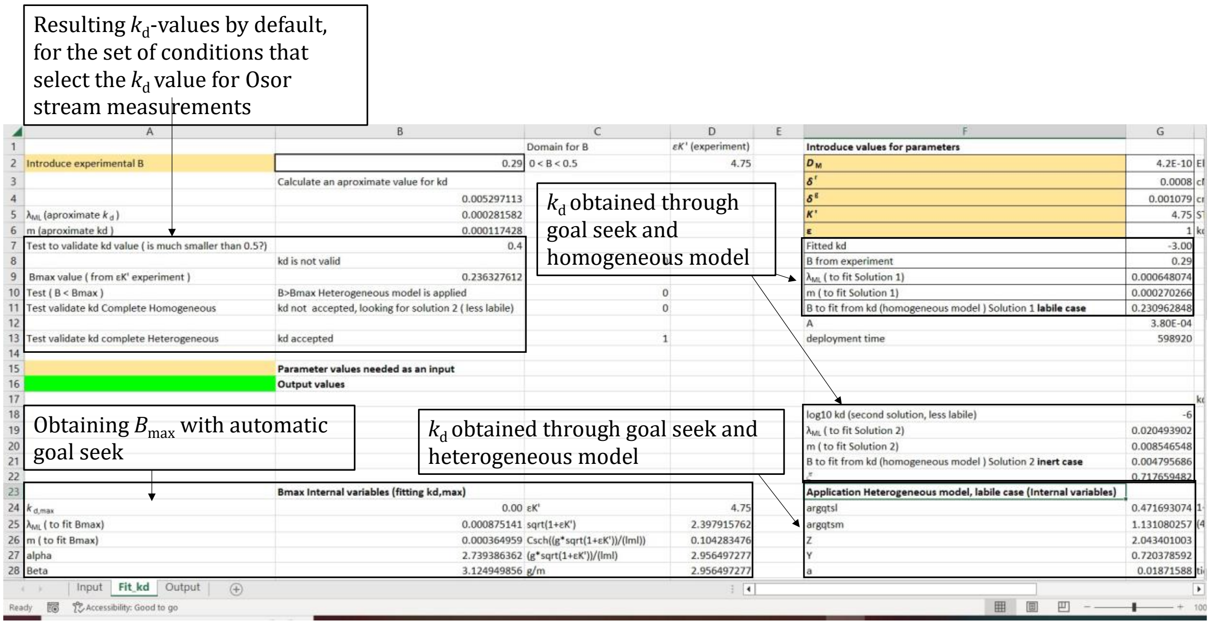

Figure S8 Screenshot of the sheet “Intermediary calculations” with a short explanation of the purpose of each section.

Figure S8 displays the internal calculations that the program does in order to fit k_d from B_{exp}. On the top left of the figure (region A7:B13), there are the set of conditions that the program automatically checks in order to select the model that gives the correct result of k_d. On the top right, it is shown the calculation of k_d with Eq (6) of the main manuscript. The computation is done twice with a different seed for the labile case (k_d=1×10^{-3} s^{-1}) and k_d=1×10^{-6} s^{-1} for the inert case. The right value is selected based on the accumulation in the DGT device. The bottom left (region A23:D28) is used to obtain the maximum B value with Eq (S94). On the bottom right (region F23:G28), the fitting of k_d is done by using Eq (S78) and the set of equations that are needed to calculate B_{het} explained in the previous sections. The fitting procedures are done by using the tool goal seek.

The sheet “Output” displays the model and the result for k_d that is obtained. The correct solution is highlighted in green.

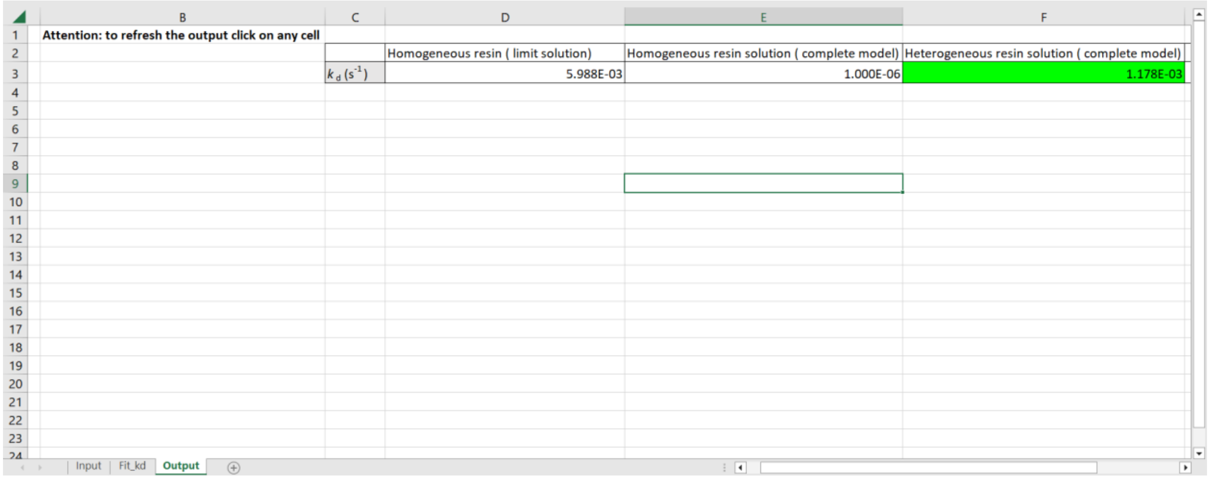

Figure S9 Output sheet of the Excel file displaying in green background the correct result for k_d.

10 Total concentrations introduced as input in VMINTEQ

Table S 1 Total concentrations of anions and trace-metals measured in Osor stream and introduced as input values in VMINTEQ for the speciation calculation.

Component Name	Total Concentration (M)
Na^+	6.25×10^{-4}
K^+	3.07×10^{-5}
Mg^{+2}	2.03×10^{-4}
Ca^{+2}	4.32×10^{-4}
Li^+	8.64×10^{-7}
NO_2^-	2.17×10^{-7}
NO_3^-	1.29×10^{-6}
Cl^-	3.42×10^{-4}
SO_4^{-2}	1.55×10^{-4}

F^-	1.47×10^{-5}
CO_3^{-2}	7.32×10^{-4}
Zn^{+2}	6.33×10^{-7}
Pb^{+2}	4.64×10^{-9}
Mn^{+2}	3.13×10^{-7}
Fe^{+2}	1.46×10^{-7}
